# Determinants of Missed Opportunities for Vaccination (MOVs) Indicators Among Children Aged 12–23 Months in Sub-Saharan African Countries: A Multilevel Analysis of Survey Data

**DOI:** 10.3390/vaccines14050417

**Published:** 2026-05-06

**Authors:** Jacques L. Tamuzi, Patrick D. M. C. Katoto, Doris Y. Sakala, Charles S. Wiysonge, Peter S. Nyasulu

**Affiliations:** 1 Division of Epidemiology and Biostatistics, Department of Global Health, Faculty of Medicine and Health Sciences, Stellenbosch University, Cape Town 7505, South Africa; katoto.chimusa@ucbukavu.ac.cd (P.D.M.C.K.); 28771907@sun.ac.za (D.Y.S.); shey.wiysonge@mrc.ac.za (C.S.W.);; 2 Cochrane South Africa, South African Medical Research Council, Cape Town 7500, South Africa; 3 Centre for Tropical Diseases and Global Health, Department of Medicine, Catholic University of Bukavu, Bukavu P.O. Box 285, Democratic Republic of the Congo; 4 Division of Epidemiology and Biostatistics, School of Public Health, Faculty of Medicine and Health Sciences, University of the Witwatersrand, Johannesburg 2193, South Africa

**Keywords:** MOV, indicators, barriers, children, 12–23 months, sub-Saharan Africa

## Abstract

**Background:** In sub-Saharan Africa, missed opportunities for vaccination are a major obstacle to reaching the global target of vaccination coverage. The study’s aim was to identify the factors driving missed opportunities for vaccination indicators, including crude missed opportunities for vaccination, all corrected, uncorrected, and some corrected missed opportunities for vaccination in children aged 12–23 months in sub-Saharan Africa. **Methods:** This was a multilevel mixed-effects population-based cross-sectional analysis using Demographic Health Surveys and Multiple Indicators Cluster Surveys data collected from 1 January 2019 to 31 December 2023 from twenty-one countries in sub-Saharan Africa. Both multilevel mixed-effects logistic regression and multilevel multinomial logistic regression were undertaken to assess the strengths of association between missed opportunities for vaccination indicators and covariates. **Results:** We included a total of 23,490 children aged 12–23 months. In multilevel fixed-effects logistic regression, our findings revealed that mothers’ education levels, listening to radio, sales ‘occupation, bicycle as mode of transportation to the nearest health facility, and health insurance were all associated with lower crude missed opportunities for vaccination. In contrast, father’s primary education and watching television were associated with increased risk of missed opportunities for vaccination. In multilevel fixed-effects multinomial logistic regression, mothers’ education levels, watching television, Muslim, and health-insured children were all associated with all corrected missed opportunities for vaccination. In the same line, mothers with primary education, watching television, private health facilities as delivery place, and health-insured children were associated with some corrected missed opportunities for vaccination. In the random-effects, the intraclass correlation coefficient showed missed opportunities for vaccination variances of 18% for crude missed opportunities for vaccination and 27% for all corrected, some corrected, and uncorrected missed opportunities for vaccination between communities in SSA. **Conclusions:** A variety of driving factors influence MOVs indicators in children aged 12–23 months, mainly caregivers’ education, media exposure, health insurance, occupation, religion, and mode of transportation to the nearest health facility. Effective evidence-based strategies are needed to minimize the influence of barriers on missed opportunities for vaccination in children aged 12–23 months in sub-Saharan Africa.

## 1. Background

Missed opportunities for vaccination (MOVs) have been identified by the World Health Organisation (WHO) as a significant factor in the world’s suboptimal immunisation coverage [1,2,3]. The WHO advises offering immunisation services at every interaction with the healthcare system in order to hasten and maintain improvements in immunisation coverage [1,2]. Despite the substantial progress made in expanding immunisation coverage globally since the launch of the Expanded Programme on Immunisation (EPI) in 1974, coverage has stalled in recent years and remains suboptimal in many countries [1,4]. The failure of sub-Saharan Africa (SSA) to immunize 90% of under five children is a significant challenge in global health, leading to health insecurity worldwide and posing a threat to other nations. MOVs has been recognized as major obstacle to attaining the desired vaccination coverage in SSA [5]. The elimination of MOVs can significantly improve immunisation coverage, thus reducing the risk of vaccine-preventable disease [6,7]. It has been proposed that increased efforts being focused on minimizing MOVs may assist countries in SSA in achieving their immunisation objectives [8]. A recent study indicated an estimated crude MOVs, all corrected, uncorrected, and some corrected MOVs of 34% [24–43%], 14% [11–18%], 15% [6–24%], and 5% [3–7%], respectively, in SSA [9]. The WHO grouped barriers driving MOVs in health facilities, caregivers, and health systems [10]. The most common MOVs barriers included maternal age, antenatal care visits, residence, educational status, high fertility rates, place of delivery, perceived healthcare support, wealth index, occupational status, media access, religious affiliation, maternal awareness of vaccination, vaccine shortages and inflexible clinic schedules, perceived contraindications to vaccination by providers and parents, negative parental beliefs regarding vaccination, insufficient inquiry by healthcare professionals about children’s vaccination status, and parity in SSA [1,6,11,12,13,14,15,16,17]. At the individual level, these factors encompass insufficient awareness of the significance of immunisation, misconceptions, and low socioeconomic status; at the health system level, they involve human resource shortages, gaps in immunisation knowledge among health facility staff, and vaccine stock-outs, among others [6,18,19,20,21].

As part of the Sustainable Development Goals, there is a growing interest in reaching “zero-dose” children, or children who have not received any routine vaccinations. This is because the goal is to not leave anyone behind when it comes to immunization [22]. However, monitoring and evaluation of vaccination in zero-dose children should be effective since they may fall into the group of MOVs. In the light of this, a recent study conducted in children aged 12–23 months revealed that factors driving zero dose were similar to MOV factors [23]. Effective MOVs assessment is an important strategy for promoting vaccination uptake among children in general, and zero-dose children especially in resource-limited settings such as SSA. In order to improve immunisation coverage, in 2017, WHO recommended a revised methodology to assess MOVs, using vaccination coverage quality indicators (VCQI) [24]. At this level, all the studies conducted on MOVs conducted in SSA have focused on MOV barriers associated with MOVs as a single indicator in children aged 12–23 months. None of them looked at barriers associated with other MOV indicators, including uncorrected, corrected, and some corrected MOVs. Furthermore, no study has been conducted to review barriers driving MOV indicators during and post coronavirus disease 2019 (COVID-19) pandemic in SSA. A study reported that the recovery from the COVID-19 pandemic will not be as same level as its initial economic ramifications, with emerging economies and disadvantaged populations needing significantly more time to recover from pandemic-induced income in SSA [25]. In view of this, many countries in SSA have still not recovered from vaccination coverage to pre-COVID-19 pandemic level, and many are becoming worse [26]. All these facts, combined with weak health systems and historical issues in immunisation systems in the majority of SSA countries, may jeopardize the global immunisation coverage target by 2030 in SSA. Knowing that MOVs are important indicators of vaccination coverage, more information on the factors driving MOVs as identified by VCQI may provide an overview of these factors and play an important role in the development and implementation of appropriate strategies to reduce MOV-related barriers and increase vaccination coverage in SSA. This study aimed at finding out factors driving MOV indicators including crude MOVs, all corrected, uncorrected, and some corrected MOVs in children aged 12–23 months and its variations across clusters in SSA.

## 2. Methods

### 2.1. Study Design

This was a multilevel mixed-effects population-based cross-sectional study, using data from the Demographic and Health Survey (DHS) and Multiple Indicator Cluster Surveys (MICS) including children aged 12–23 moths in SSA from 1 January 2019 to 31 December 2023.

### 2.2. Data Sources

The DHS and MICS initiatives are ongoing collaborations between the United States Agency for International Development (USAID), United Nations International Children’s Emergency Fund (UNICEF), as well as country-specific organizations. In low- and middle-income countries (LMCs), they undertake nationally representative household sample surveys on a variety of population health indicators [27,28]. The DHS and MICS were collected using comparable nationally representative household surveys from 1 January 2019 to 31 December 2023. Data from DHS and MICS are gathered using four main questionnaires [27,28]. The household survey gathers data on household attributes and enumerates all household members. The household questionnaire gives a brief overview of each household’s most important traits. It is used to choose men and women for one-on-one interviews [11]. Children under the age of five are frequently among eligible household members [27,28].

### 2.3. Participants

We considered all DHS and MICS with available individual-data records of children aged 12–23 months in SSA published from the 1 January 2019 to 31 December 2023. Surveys were selected based on the immediate pre-COVID-19 pandemic picture of MOVs and post COVID-19 pictures in SSA.

#### 2.3.1. Inclusion Criteria

In this study, we included children aged 12–23 months with available vaccination status recorded in DHS and MICS from 1 January 2019 to 31 December 2023. In case two or more surveys were conducted in the same country between 1 January 2019 and 31 December 2023, the surveys merged them to constitute one survey. Only DHS and MICS undertaken in SSA were included. Fully vaccinated (basic antigens) children aged 12–23 months defined as those who were administered for Bacillus Calmette-Guérin (BCG), 3 doses of diphtheria, tetanus and pertussis vaccine (DPT)-containing vaccine, 3 doses of polio vaccine (excluding polio vaccine given at birth), and 1 dose of measles-containing vaccine (MCV) were included [27]. In addition, we also included children who received three doses of pneumococcal conjugate vaccine (PCV) and two doses of rotavirus vaccine, on top of being fully immunised children (FIC).

#### 2.3.2. Exclusion Criteria

From the study design, we excluded children aged 12–23 months with reasons, mainly unknown vaccination status, including no vaccination card or related document, vaccination card not seen, and no longer has vaccination card. From the analysis, children aged 12–23 months with dates that are obviously wrong, mainly vaccination before birth, vaccination after the survey, vaccination series dates out-of-order or series with the same date for consecutive doses were excluded.

### 2.4. Data Collection and Sample Size Procedures

This study’s data collection and management has been published elsewhere [9]. Multistage stratified sampling procedures were used to produce a sample of children aged 12–23 months. A two-stage sampling selection approach is used in the survey methodology. First, geographical areas are chosen at random, taking into account the probability of selection [27]. The fieldwork teams then visit the designated locations to acquire a complete list of houses and households. Each country’s sample size was proportional to its population, and the sample were stratified within countries by urban and rural residence [27]. Primary sampling units (PSUs) were selected within parcels based on population-related probability and were villages or groups of villages in rural areas and enumeration blocks in urban areas [13]. Households were chosen at random from PSUs. All children under the age of five were included in selected homes. DHS and MICS require the use of sample weights during analysis due to the multistage sampling design [13,14]. The DHS and MICS respondents are chosen in two stages, stratified by urban and rural area. The DHS and MICS populations are indicative of the entire country or region of interest. In the DHS and MICS, standard data collection techniques and interviewer training ensure that the data is both reliable and comparable. This analysis makes use of the most recent DHS and MICS data surveys conducted in twenty-one countries in SSA published from 1 January 2019 to 31 December 2023. If a country has multiple eligible surveys, we merged them to obtain one survey. In this study, we use DHS and MICS data on routine immunisation among children aged 12–23 months. Variables related to caregivers and health systems data were collected. The country sample size was computed as follows: Benin (1828), Burkina Faso (1035), Chad (1154), Comoros (652), Cote d’Ivoire (806), Eswatini (367), Gabon (780), Gambia (759), Ghana (899), Kenya (2608), Liberia (338), Madagascar (658), Malawi (2519), Mozambique (564), Nigeria (3083), Rwanda (753), São Tomé and Principe (317), Sierra Leonne (731), Senegal (1789), Tanzania (852), and Zimbabwe (1789). Table 1 described the surveys and total weighted sample size of children aged 12–23 included in this study.

### 2.5. Outcomes and Definitions

The study outcomes included childhood MOVs vs. no MOVs (binary outcome) and uncorrected, all corrected, and some corrected MOVs among children aged 12–23 months (categorical outcome). All corrected MOVs are defined as children who had not received the vaccine(s) by the time of the survey, divided by the number of children who had at least one recorded vaccination date and were eligible for at least one vaccine [9,24]. Uncorrected MOVs refer to children who, by the time of the survey, had not received the vaccine(s), calculated using the same denominator: children with at least one vaccination date recorded who were eligible for at least one vaccine [9,24]. Some corrected MOVs specifically refer to children in the numerator who received some, but not all, of the recommended vaccine(s) at a later visit, as shown on the vaccination card, divided by the same group of eligible children with at least one vaccination date recorded [9,24]. VCQI software version 1.05 generated those outcomes of each child by analyzing the dates of all received vaccine doses relative to the recommended schedule and the date of the survey.

### 2.6. Explanatory Variables

The following explanatory variables were drawn from the DHS and MICS: gender of child, age of mother, age of household head, household number, residence, number of children under five, sex of household head, caretaker/mother education, father’s education, occupation, religion, wealth index quintile, means of transportation, household exposure to media, visited health facility in the last twelve months, health insurance coverage, vaccination location, reason for not using health facility/costs too much, place of delivery, distance to health facility. Definitions and categories of explanatory are described in the Supplemental Table S1.

### 2.7. Statistical Analysis and Model Building

Due to the multistage sampling criteria of DHS and MICS, the design effect parameters using svyset syntax “sampling weight,” “sampling area,” and “sampling cluster” were included in all analyses. This process involves handling survey design variables and selecting suitable fixed effects or random intercepts to appropriately account for variations at the country level. We generated weight and set survey design parameters. Exploratory data analysis described the characteristics of the study population and were expressed in proportions. Frequency tabulations were produced for caregivers’, health workers, and health systems variables. Based on VCQI analysis, indicators for crude MOV, all corrected, and uncorrected MOVs were generated. MOVs vs. no MOVs and all corrected, some corrected, and uncorrected MOVs variable were dependent variables. Prior to regression analysis, multicollinearity between explanatory variables using variance inflation factor (VIF) was used. Healthcare barriers and caregiver knowledge and engagement were removed from further analysis because of small sample size that may include outliers. In addition, we found that there was no multicollinearity between the rest of the variables. Bivariable analysis and χ^2^ tests were employed to evaluate the association between MOVs and non-MOVs, alongside covariates, to derive the crude odds ratios. We used bivariable analysis to choose the right variables for the multilevel logistic regression analysis, which we then did. The theory-based set of covariates, including maternal education, wealth, media exposure, delivery place, and insurance, was chosen according to well-established conceptual frameworks of social determinants of health. These covariates typically encompass a mix of socioeconomic, demographic, and health access variables. This theory was employed to retain and integrate factors in multilevel mixed-effects logistic regression analysis utilising both fixed and random effect models. A multilevel mixed-effects multinomial logistic regression analysis, which includes both fixed and random effect models, was used to explore the association between uncorrected, corrected, and some corrected MOVs and covariates. To estimate the model, a Generalized Structural Equation Model (GSEM) with a logit link function was fitted using Stata 19 MP’s gsem command. The adjusted relative risk ratio (ARRR) in the multivariable study was identified as a significant predictor of all corrected and some corrected MOVs. We calculated the unadjusted as well as adjusted association between outcomes and explanatory variables by reporting the odd ratios (95%CI) and RRR (95%) for each type of regression included in this study. The *p*-value of <0.05 was considered as statistically significant. Because of the progressive information structure of DHS and MICS data, we expect unmeasured impacts at the district, community, and person levels. Then, the variability of MOVs and no MOVs across clusters, communities, and countries was evaluated using the community-level variance, intraclass correlation coefficient (ICC), proportional change in variance (PCV), and Median Odds Ratio (MOR) in a random-effects analysis (see Equations (1)–(3)). Furthermore, we also computed the predicted posterior mean (95%CI) using random-effects post-estimation of the best. Four different nested models were built. The models were null model (containing only the outcome variable), Model 1 (corrected MOV status and caregivers’ variables), Model 2 (corrected MOV status and health system variables), and Model 3 (corrected MOVs status, caregivers’, and health system variables). The final interpretation of results was based on the best model among them, which is selected using the log-likelihood ratio (LLR), Akaike’s Information Criterion (AIC), and Bayesian Information Criterion (BIC). To maximise the plausibility of the missing-at-random assumption, we produced 20 imputed datasets using chained equations that included all study variables and the main outcome. Each imputed dataset was examined separately, and parameter estimates were pooled according to Rubin’s guidelines [29], allowing us to account for the uncertainty provided by missing data and evaluate the robustness of our findings. We conducted a sensitivity analysis including random-effects by country vs. conditional logistic models fixed-effects models the robustness of the direction and magnitude of your core independent variable were stable. Stata 19.5 MP and GrapPad Prisma 9.2.0 software were used for all analyses.

(1) $$ICC=\partial^{2}/(\partial^{2}+\frac{\pi^{2}}{3})$$

(2) $$PCV=\frac{var\left(null\;model\right)-var(full\;model)}{var(null\;model)}$$

(3) $$MOR=esp(\sqrt{2\times \partial^{2}\times 0.6745\;})$$

$\partial^{2}$ indicates the cluster variance, and PCV quantifies the overall variation attributable to individual and community-level components in the multilevel model relative to the null model. MOR quantifies the diversity or heterogeneity in outcomes among clusters and is defined as the median value of the odds ratio between the cluster with a high likelihood of MOVs and MOVs correction status, and the cluster with a lower likelihood when randomly selecting two clusters.

### 2.8. Ethical Approval

The study used publicly available secondary data from the DHS (https://dhsprogram.com/ accessed on 12 October 2024) and MICS (http://mics.unicef.org/surveys accessed on 24 November 2024). Children as well as caregivers were not involved in the design and conduct of this research. Ethical approval to use these data was obtained from DHS and MICS projects. The DHS and MICS initiatives provided ethical permission for the use of this data. In addition, the Health Research Ethics Committee of the Faculty of Medicine and Health Sciences at Stellenbosch University approved this research (Reference Number: S25/08/208).

## 3. Results

### 3.1. Description of Potential Factors Driving MOV Among Children Aged 12–23 Months in Sub-Saharan Africa

In Supplemental Table S2, we calculated the VIF and 1/VIF for each variable included in the analysis, as well as the mean VIF for all variables. The variable for children’s gender exhibited the lowest VIF at 1.02, whereas the wealth index displayed the greatest VIF at 1.89.

A total weighted sample of 23,490 children aged 12–23 months from twenty-one countries in SSA were included. Among them, 51.08% were boys and 48.92% were girls (Supplemental Table S3). Households with men as heads had higher proportion (78.58%) than females (21.42%) (Supplemental Table S3). Household size of six and above reported higher proportion (55.63%) than those with 4 to 5 or 1–3 people (Supplemental Table S3). The proportion of households with one child under 5 (42.73%) was less than those with two children (48.70%). More than half of households (55.63%) had six or more members. Our results also showed that the proportion of women aged 15–24 years (55.43%) was greater than those aged 25–34 years (31.81%) and 35–49 years (12.77%). About mother’s/caretaker’s education, the proportions of not educated, primary, secondary, or higher levels were 33.61%, 31.31%, and 35.08%, respectively (Supplemental Table S3). Husbands/partners education without education, primary, secondary, or higher levels were 36.53%, 24.75%, and 33.18% (Table 2). Most of the children aged 12–23 months resided in rural area (67.83%) vs. urban (32.17%). Around 37.96% of heads of household were unemployed (Table 2). About the wealth index, both the poorest and poor households reported the proportions of 25.85% and 22.18%, respectively (Supplemental Table S3). About religion, other Christians, including Pentecostal, apostolic, evangelical, revival, Zion, African instituted, and others, reported 35.43%, higher than Muslim (35.52%) and Catholics (10.12%) (Supplemental Table S3). Regarding media access, the proportions of households with internet access, reading newspapers/magazines, listening to radio, and watching television were 27.43, 11.45%, 49.82%, and 48.76%, respectively (Supplemental Table S3).

Our study showed that around 75.81% of children aged 12–23 months were born in the public health facilities (Supplemental Table S3). In the same line, 84.85% % of children aged 12–23 months were vaccinated in the public health facilities (Supplemental Table S3). In addition, only 12.02% of households had health insurance (Supplemental Table S3). About distance to health facilities, 63.88% of households reported that this was not a big problem (Supplemental Table S3). The reason for not using the health facility was not too far, being reported at 99.23%, and not too costly at 2.41% (Supplemental Table S3). In addition, 74.39% of caretakers reported that they visited health facility in the last 12 months (Supplemental Table S3).

### 3.2. Description of Potential Factors Driving Uncorrected, Corrected, and Some Corrected MOV Among Children Aged 12–23 Months in Sub-Saharan Africa

In the total of 7649 children aged 12–23, 50.58% were boys and 49.42% were girls (Supplemental Table S4). Roughly a third of household heads were males, and almost half of households had two to three children under five years (Supplemental Table S5). In the same line, approximately a quarter of household heads was above 50 years, and 58.11% of households had more than six members (Supplemental Table S4). Among women 15–49 years, approximately half of them were aged 15–24 years (Supplemental Table S4). Descriptive analysis also showed that 36.85 of children’s mothers lacked education and 38.44% of husbands/partners also lacked education (Supplemental Table S4). About marital status, 83.18% of women were married or living with a partner (Supplemental Table S4). Our results also revealed that approximately a third of children aged 12–23 months resided in rural areas (Supplemental Table S4). The findings also showed that 44.33% of household heads were jobless and 28.38% of households are classified as the poorest wealth index quintile (Supplemental Table S5). Other Christians and Muslims were the most dominated religion with 34.57% and 34.31%, respectively (Supplemental Table S4). About media access, 26.01%, 11.33%, 43.16%, and 47.68% of households had access to the internet, read newspapers or magazines, listen to radio, and watch television, respectively (Supplemental Table S4).

Our finding showed that 72.69% of children were delivered in public health facilities and 87.79% were vaccinated in public health facilities (Supplemental Table S4). In the same line, 11.78% of households had health insurance (Supplemental Table S5). Our findings also revealed that 41.56% of households reported that distance to health facility was a big problem, only 0.50% reported that reason for not using health facilities was because of lack of access and too far, and 4.76% reported that reason for not using health facility because of higher costs (Supplemental Table S4). Roughly 90.51% of households immunised their children aged 12–23 months in the government or public health facilities (Supplemental Table S4).

### 3.3. Multilevel Fixed-Effects Logistic Regression Analysis in Assessing Factors Associated with MOV Among Children Aged 12–23 Months in Sub-Saharan Africa

In bivariable logistic analysis, our results showed that an increased risk of MOVs in households with two to three children (COR 1.20, 95%CI 1.11–1.29) and four and above children under five (COR 1.26, 95%CI 1.07–1.47) (Table 2). In the same line, household with six members and above reported an increased risk of MOVs (COR 1.16, 95%CI 1.04–1.30) and rural residence (COR 1.48, 95%CI: 1.11–2.08). A decreased risk of MOVs was also observed for households that were watching television (COR 0.88, 95%CI 0.80–0.97), listening to radio (COR 0.63, 95%CI 0.59–0.68), and using internet (COR 0.83, 95%CI 0.76–0.91) (Table 2). In the same line, mothers with primary (COR 0.84, 95%CI 0.77–0.91), secondary, or higher education (COR 0.75, 95%CI 0.68–0.83) were at lower risk of MOVs (Table 2). In point of view occupation, children aged 12–23 months from parents who were agriculture (COR 0.61, 95%CI 0.51–0.73), manual, house helper, and domestic occupations (COR 0.82, 95%CI 0.69–0.97), sales (COR 0.51, 95%CI 0.45–0.58) and professional, technical, managerial, clerical, and service occupations (COR 0.81, 95%CI 0.69–0.94) were at lower risk of MOVs (Table 2). Our findings indicated a decreased risk in MOVs among children aged 12–23 months based on the household wealth index, with a COR of 0.88 (95%CI 0.79–0.98) for middle wealth, a COR of 0.85 (95%CI 0.76–0.96) for richer, and 0.79 (95%CI 0.70–0.89) for richest wealth (Table 2). Our analysis also showed that the religion of the household heads, including other Christians (Pentecostal, apostolic, evangelical, revival, Zion, African instituted, and others) (COR 0.72, 95%CI 0.63–0.83), Protestant (COR 0.58, 95%CI 0.49–0.69), and Islam (COR 0.70, 95%CI 0.60–0.81) were at decreased risk of MOVs in their children aged 12–23 months (Table 2).

Regarding health system barriers, the place of delivery such as public health facilities (COR 0.69, 95%CI 0.60–0.79), private health facilities (COR 0.65, 95%CI 0.49–0.86), and NGO/Religious (COR 0.62, 95%CI 0.41–0.95) were at lower risk of MOVs for children aged 12–23 months. Caretakers who visited health facilities in the last 12 months had a lower risk of MOVs for their children aged 12–23 months (COR 0.73, 95%CI 0.64–0.83) (Table 2). In contrast, households that did not access health facilities because long distance reported an increase MOVs in their children aged 12–23 months (COR 1.47, 95%CI 1.31–1.64) (Table 3). About the distance to health facility, households that reported that this was a big problem were at increased MOVs risk of COR 1.47, 95%CI 1.31–1.64 (Table 2). Households that reported that they could not access healthcare because it was too costly were at greater risk of MOVs (COR 7.22, 95%CI 3.39–15.37) (Table 2). Furthermore, households that arrived at the closest health facility within an hour and two hours were more likely to have MOVs for their children aged 12–23 months, with COR (95%CI) of 1.19 (1.03–1.37) and 1.70 (1.35–2.15), respectively (Table 2).

**Figure 1:**
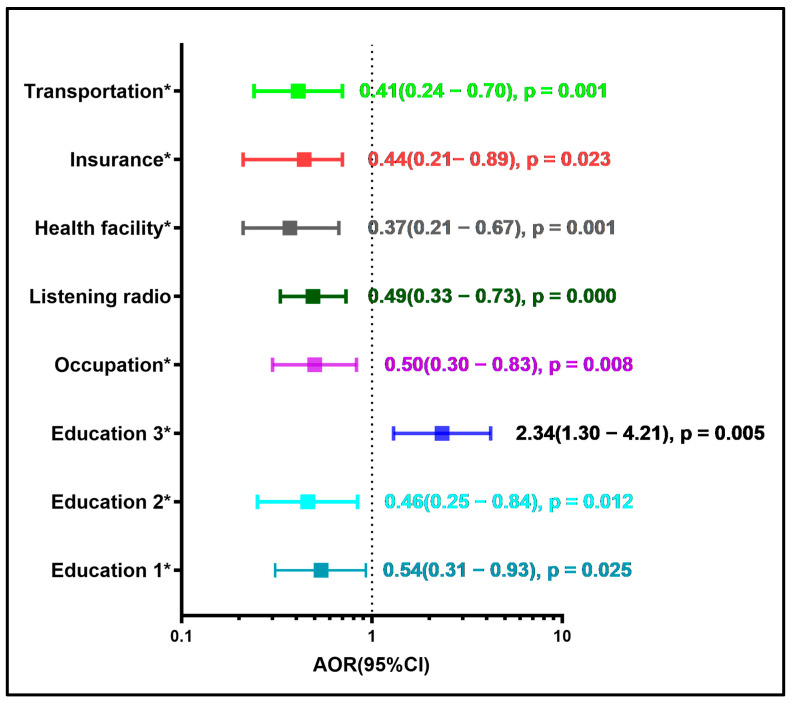
Multivariable analysis of factors associated with missed opportunities for vaccinations in children aged 12–23 months in sub-Saharan Africa. * Note: Education 1 *: Mothers’ primary education; Education 2 *: Mothers’ secondary or higher education level; Education 3 *: Father’s primary education level; Health facility *: Public health facilities as place of delivery; Insurance *: Health insured children; Transportation *: Mode of transportation to the nearest health facility (bicycle/animal-drawn/boat no motor).

In multilevel fixed-effects logistic regression analysis, mothers with primary educational level (AOR 0.54, 95%CI 0.31–0.92) and secondary and higher (AOR 0.46, 95%CI 0.25–0.84) had a reduced risk of MOVs for their children aged 12–23 months (Table 2 and Figure 1). In the same line, households that were listening to the radio were at lower risk of MOV for their children aged 12–23 months (AOR 0.49, 95%CI 0.33–0.73) (Table 2 and Figure 1). Mode of transportation including bicycle, animal-drawn, and boat with no motor showed a lower risk of MOVs (AOR 0.41, 95%CI 0.24–0.70) (Table 3 and Figure 1). Sales’ occupation was at lower risk of MOVs (AOR 0.50, 95CI 0.30–0.83). In contrast, fathers with primary educational level (AOR 2.34, 95%CI 1.30–4.21) had an increased risk of MOVs for their children aged 12–23 months (Table 2 and Figure 1). Regarding health system barriers, the public health facilities were at lower risk of MOVs for children aged 12–23 months (AOR 0.37, 95%CI 0.21–0.67). In the same line, households with health insurance showed a lower risk of MOVs (AOR 0.43, 95%CI 0.21–0.87) (Table 2 and Figure 1). After multiple imputation, all the variable before imputation remained statistically significant accept father’s education (Supplemental Table S5). This finding should be interpreted in the context of confounding that the model could not control. In the same line, multiple imputation revealed that agriculture (AOR 0.50, 95%CI 0.33–0.78) and professional, technical, managerial, and clerical occupations (AOR 0.78, 95%CI 0.62–0.98) had a reduced risk of MOVs for their children aged 12–23 months (Supplemental Table S5). In contrast, households with time to reach the nearest health facility of one hour (AOR 1.35, 95%CI: 1.14–1.61) and two hours (AOR 1.55, 95%CI 1.14–2.11) (Supplemental Table S5). When we looked at directional and magnitude stability in resilience of random-effects vs. fixed-effects conditional logistic models (Supplementary Table S6), the sensitivity analysis revealed that our results were not robust at the country level.

### 3.4. Multilevel Fixed-Effects Multinomial Logistic Regression Analysis Assessing Factors Associated with MOV Correction Status Among Children Aged 12–23 Months in Sub-Saharan Africa

Among the four models built, we reported Model 3 which best fitted to our data. Before conduction of multilevel fixed-effects multinomial logistic regression analysis, a bivariable multinomial logistic regression analysis assessing factors associated with MOVs correction was undertaken (Supplemental Table S7). Null Model, Models 1, and 2 were reported as Supplemental Table S8. GSEM utilized to build Models 1, 2, and 3 were shown in Supplemental Figures S1–S3.

A multilevel fixed-effects multinomial logistic regression analysis was fitted to assess the factors associated MOVs correction status among children aged 12–23 months in SSA. MOVs correction status was classified as corrected, partially corrected, or uncorrected MOVs. Uncorrected MOVs was considered the basic category.

**Figure 2:**
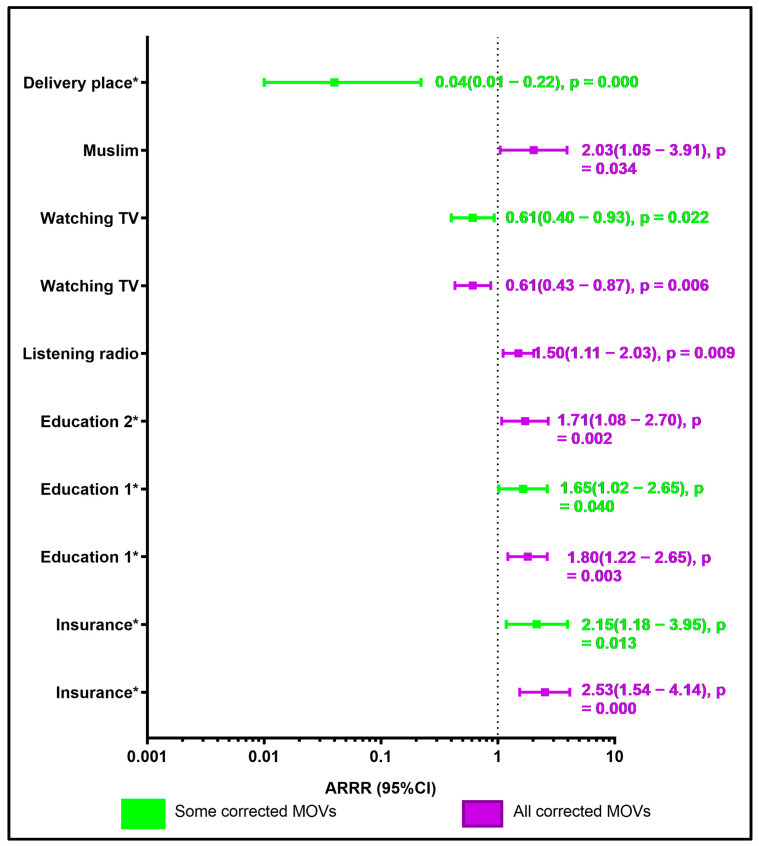
**Factors associated with correction and some correction MOV in children aged 12–23 months in sub-Saharan Africa** * Note: Insurance *: Health-insured children; Education 1 *: Mother’s primary education; Education 2 *: Mother’s secondary or higher education level; Vaccination place: NGO/Religious health facilities; Delivery place *: Private health facilities.

**Factors associated all corrected MOVs:** caretaker’s primary education (ARRR 1.80, 95%CI 1.22–2.65) and secondary or higher education (ARRR 1.71, 95%CI 1.08–2.70) were more likely to have all corrected MOVs for their children aged 12–23 months (Table 3 and Figure 2). Our findings also showed that Muslim were more likely to have all corrected MOVs for their children aged 12–23 months (ARRR 2.03, 95%CI 1.05–3.91). Households that were listening to the radio were 1.50 times more likely to all corrected MOVs for their children aged 12–23 months (ARRR 1.50, 95%CI 1.11–2.03). In contrast, households that watched television were less likely to all corrected MOVs for their children aged 12–23 months (ARRR 0.61, 95%CI 0.43–0.87) (Table 3 and Figure 2). Lastly, households with health insurance were 2.53 times more likely to all corrected MOVs for their children aged 12–23 months (ARRR 2.53, 95% 1.54–4.14) (Table 3 and Figure 2).

**Factors associated with some corrected MOVs**: Our results revealed that mothers with primary education level were 1.65 times more likely to have some corrected MOVs for their children aged 12–23 months (ARRR 1.65, 95% 1.02–2.65) (Table 3 and Figure 2). In contrast, household watching TV had the risk reduction of 39% for some corrected MOVs for their children aged 12–23 months (ARRR 0.61, 95% 0.40–0.93) (Table 3 and Figure 2). Our findings also showed that households with health insurance were 2.15 times more likely to have some corrected MOVs for their children aged 12–23 months (ARRR 2.15, 95%CI 1.17–3.95) (Table 3 and Figure 2). Children aged 12–23 who were vaccinated in NGO/conventional health facilities were less likely to have some corrected MOVs (Table 4 and Figure 2).

### 3.5. Models’ Fitness of Factors Associated with MOVs Indicators in SSA

Table 4 showed that models 3 had the highest LLR, and lowest AIC and BIC in both multilevel logistic regression analysis (LLR = −514.58, AIC = 1117.16, and BIC = 1332.92) and multilevel multinomial logistic regression analysis (LLR = −1340.88, AIC = 2785.77, and BIC = 3060.22) and were the best-fitted models for these two types of analysis.

### 3.6. Random-Effects Model Assessing Factors Associated with MOVs Indicators in SSA

For random-effects models, the ICC for the best model was estimated at 18% for multilevel logistic regression analysis and 27% for multilevel multinomial logistic regression analysis (Table 5a,b). PCV illustrated that seventy-six percent of the variability in MOVs and sixty-five of the variability in MOVs correction were explained by Model 3, with the residual variance possibly attributable to unquantified variables or intricate relationships (Table 5). A child in a cluster with a high probability of MOVs had 2.37 times greater odds of having MOVs compared to a child in a cluster with a low probability of MOVs during the random selection of children aged 12–23 months from two distinct clusters (Table 5). Additionally, a child in a cluster with a high probability of corrected and some corrected MOVs had 2.58 times greater odds of having corrected and some corrected MOVs compared to a child in a cluster with a low probability of all corrected and some corrected MOVs during the random selection of children aged 12–23 months from two distinct clusters (Table 5).

**Figure 3:**
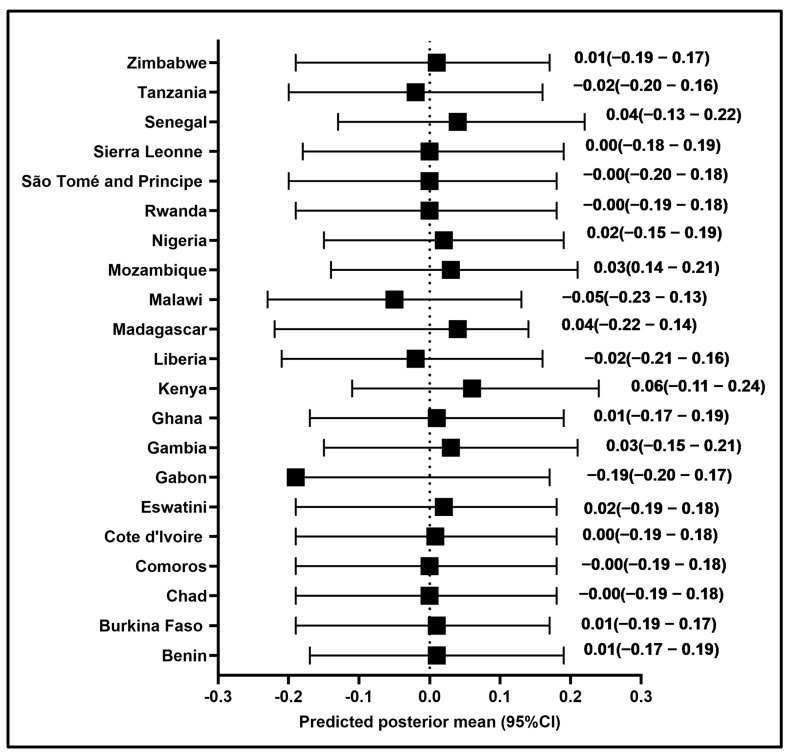
Predicted posterior mean (95%CI) of MOVs in children aged 12–23 months using Model 3 by country in SSA.

Using multilevel post-estimation, the average (95%) of MOVs vary over time with random effects using Model 3. Based on Model 3, Figure 3 depicts the change in predicted posterior means (95%CI) of my MOVs over time with random effects for twenty-one sub-Saharan African countries. In Malawi, children aged 12–23 months had an average probability of having MOVs of 0.05 (95%CI: −0.23 to 0.13), whereas in Madagascar it was 0.04 (95%CI: −0.22 to 0.14) (Figure 3 and Supplemental Table S9). In contrast, children aged 12–23 months in Mozambique, Senegal, and Kenya had an average probability of having MOVs that decreased by 0.03 (95%CI: −0.14 to 0.21), 0.04 (95%CI: −0.13 to 0.21), and 0.06 (95%CI: −0.11 to 0.23), respectively (Figure 3 and Supplemental Table S8). Gabon was emphasised since the model indicates that observations would have a negative predicted posterior mean of MOVs (−0.19, 95%CI: −0.20 to 0.17) (Figure 3 and Supplemental Table S8). However, This Figure 3 indicated that no countries exhibited outliers while accounting for our model’s uncertainty. Similarly, none of the 95%CIs of predicted posterior means were statistically significant, showing that the predictor’s effect was not significantly different in at least one country.

Supplemental Table S10 depicts the model-predicted probability of uncorrected, corrected, and some corrected MOVs in multinomial logistic regression analysis of key variables, showing the likelihood of key variables falling into each of MOVs correction categories (uncorrected, corrected, and some corrected MOVs).

## 4. Discussion

This study’s aim was to find out barriers driving MOVs indicators, including crude MOVs, all corrected, uncorrected, and some corrected MOVs in children aged 12–23 months and its variations across countries and provinces in SSA by using DHS and MICS data. Data were collected from twenty-one sub-Saharan African countries, including Benin, Burkina Faso, Chad, Comoros, Cote d’Ivoire, Eswatini, Gabon, Gambia, Ghana, Kenya, Liberia, Madagascar, Malawi, Mozambique, Nigeria, Rwanda, São Tomé, Sierra Leone, and Príncipe, Senegal, Tanzania, and Zimbabwe.

After confounding adjustments, multilevel fixed-effects logistic regression analysis showed that mothers’ education level of primary and secondary or higher, listening to ratio, sales ‘occupation, mode of transportation such as bicycle, and households with health insurance and place of delivery in the public health facilities showed a lower risk of MOVs for their children aged 12–23 months. In contrast, fathers’ primary education level was at greater risk of MOVs for their children aged 12–23 months. In multilevel fixed-effects multinomial logistic regression, our results revealed that caretakers/mothers with primary, secondary or higher education, Muslim as the head household ‘religion, and households with health insurance were more likely to have all corrected MOVs for their children aged 12–23 months. In contrast, about some corrected MOVs, our findings showed that mothers’ education level of primary and households with health insurance were more likely to have some corrected MOVs for their children aged 12–23 months. However, households who had delivery of a baby in private health facilities were less likely to have some corrected MOVs for children aged 12–23 months. In addition, limited media access, including listening to the radio and watching television may either increase or decrease MOVs indicators.

Based on the study’s findings, mothers’ education, listening to the radio, health-insured children, and public health facilities as delivery places played a key role in throughout the pathway of reducing or increasing MOVs indicators in children aged 12–23 months in SSA (Figure 1 and Figure 2). Compared to other studies, our results were similar to those conducted in SSA that showed that mothers’ education level plays an important role in minimizing MOVs in childhood [1,30,31,32]. According to a recent meta-analysis, children whose mothers had primary education were 1.87 times more likely to receive all recommended childhood vaccinations than those whose mothers had no education [33]. Various studies have found that mothers with higher levels of education are more likely to be aware of vaccine-preventable diseases, understand the benefits of immunization, and ensure their children complete vaccinations on schedule [33,34,35,36,37,38]. On the other hand, mothers without formal education may lack adequate knowledge about their child’s vaccinations, proper childcare, and overall health. Maternal education is vital in shaping positive attitudes and perceptions toward child health [33,39,40,41,42]. According to this study’s descriptive analysis, uneducated mothers had a greater rate (37.0%) than those with primary, secondary, or higher education. Furthermore, approximately half of the mothers were between the ages of 15 and 24 years. Targeting the age group of uneducated mothers aged 15–24 years may play an important role in enhancing MOVs in SSA. A study has shown that educating mothers in this age group can help reduce and avoid vaccine-preventable infections and related risks in SSA [33]. Our findings were also in the line with a study that showed that children whose fathers had primary education were less likely to have vaccination uptake [43]. Similarly, a study found that compositional and structural characteristics explain education-related inequalities in MOVs in children under the age of five [44]. Indeed, correcting all MOVs is crucial for preventing further outbreaks of vaccine-preventable diseases and sustaining herd immunity among children under five. Therefore, it is essential for parents to ensure their children receive vaccinations promptly and consistently. To achieve this, healthcare providers should focus on building vaccine confidence by educating and empowering parents through clear and understandable communication. A study showed that healthcare professionals continue to be the most trusted source of information for parents [45]. To enhance MOV indicators in SSA, routine prenatal and postnatal care should include a vaccine chat with mothers and fathers, which would inform and promote decision-making while also addressing concerns.

A significant association was found between households holding health insurance and lower risk of MOVs in children aged 12–23 months. Studies have shown that children who had never been insured had significantly lower vaccination coverage than children who had been insured continuously [46]. Another study reported that health insurance produced substantial increases in the likelihood of childhood vaccination [47]. Knowing that health spending in primary healthcare in SSA remains low, the implementation of health insurance programmes to foster accessibility to healthcare with focused on increasing health insurance coverage for low-income children’s households. Health insurance may still prove to be an effective mechanism for removing financial barriers to accessing vaccination in under five in SSA and protecting households against costs of healthcare. According to studies, children who have health insurance are more likely to obtain higher vaccination uptake than uninsured children [48,49]. However, this is likely to require wide health system strengthening efforts to improve the quality and accessibility of health services in SSA [50]. From our descriptive analysis, only 11.74% of households are covered by medical insurance for their children aged 12–23 months in SSA. This analysis revealed that medical insurance accessibility remains low in SSA. Compared to home delivery, public health institutions had lower MOVs and fewer some corrected MOVs. Our findings were consistent with research published in SSA that indicate that MOVs in children are more frequent following home births than health facility deliveries, indicating a relationship between the location of delivery and vaccination coverage [51,52]. With an estimated 19.49% of home deliveries in SSA, as demonstrated in our descriptive analysis, interventions should concentrate on reducing obstacles to healthcare access and encouraging facility-based delivery. These initiatives might significantly increase vaccine uptake and reduce MOVs in children aged 12–23 months in SSA.

Another significant predictor of MOVs indicators among children aged 12–23 months was households that accessed media, including listening to the radio. In fact, media influences vaccination positively or negatively, inducing hesitant or resistant to vaccinating children. Studies found media access was among the largest contributors to inequality in MOVs in under-five children [30,31,53]. Listening to radio may reduce MOVs and enhance all corrected MOVs or some corrected MOVs in children aged 12–23 months in SSA. In fact, vaccine hesitancy poses a significant global health challenge, fuelled by misinformation and anti-vaccination campaigns on social media platforms. In contrast, watching television decreased the risk of all corrected and some corrected MOVs; we hypothesized that inaccurate information about vaccination in children aged 12–23 months was broadcast to the caretakers, and this increased misinformation during COVID-19 pandemic. UNICEF indicated that during the COVID-19 pandemic, Sub-Saharan Africa saw a substantial “info-demic,” characterised by elevated levels of vaccination misinformation that directly influenced vaccine hesitancy and reduced uptake [54].

Vaccine hesitancy is a complex issue that is influenced by a variety of individual and cultural factors, including religious beliefs, which are typically linked to greater levels among populations. In addition, our results contrasted with previous studies that showed that Muslim religion was associated with lower vaccine coverage in several SSA countries [55]. Understanding the context of religion in the lives of community members is crucial to comprehensively evaluate its role in shaping vaccine attitudes and behaviours. In comparison to caregivers walking to reach the nearest health facilities, those using bicycle had a reduced risk of crude MOVs. Our results were in the line with other studies that have shown that long distance walking to the nearest health facility increased MOVs [17]. This might be attributed to the fact that walking distance serves as a substantial impediment to timely access to healthcare services, particularly in remote areas. Our results also showed that sales’ occupation was associated with lower risk of MOVs. A study showed higher MOVs prevalence estimated among children of caregivers employed as sales [32]. However, this should be seen in angle of contextual-associated factors associated with some corrected MOVs in SSA.

Bivariable analysis showed a decreased risk in MOVs among children aged 12–23 months based on the household wealth index for poor, middle wealth, richer, and richest wealth compared to the poorest households. This was in the line with studies that revealed that rich children were at an advantage compared to the poor in terms of vaccination uptake [31,56,57]. In addition, distance to the nearest health facility as a big problem, long distances to the nearest health facilities, and costly healthcare access were at greater risk of MOVs for their children aged 12–23 months. A study revealed that healthcare access in SSA stands at just 42.56%, which remains quite low despite Sustainable Development Goal 3.8′ s aim of achieving universal health coverage to ensure that everyone can access the necessary health services [58]. These inequalities underscore the difficulties that low-income households encounter in obtaining vaccinations for their children under five. [31,56,57]. Our results also revealed that the magnitude of MOVs indicators varied from communities across SSA as shown by computed ICC, community-level variance, PCV, and MOR. This could be explained by differential influences such as socioeconomic positions, media availability, education-related inequalities, and immunisation locations associated with MOVs indicators.

Interventions to reduce gaps in education-related inequality in MOVs should focus on social determinants of health and are needed in SSA [44]. Effective interventions have been explored to minimized MOVs. Additionally, several strategies can be considered to minimize MOVs among children aged 12–23 months by enhancing different components of immunization programs. These interventions include promoting facility-based deliveries through programs such as Integrated Postnatal Care and Integrated Management of Childhood Illness (IMCI); expanding insurance coverage by incorporating national health insurance schemes; and using targeted radio campaigns that leverage local media to broadcast educational messages in local languages, ensuring mothers and caregivers know when and where to immunize their children. Other interventions involve implementing reminder and recall systems like electronic immunization registers, as well as leveraging artificial intelligence (AI) to identify underserved populations, forecast vaccine demand, automate reminders, and address vaccine-related misinformation. Furthermore, communication strategies targeted at caretakers, health workers, and religious leaders [59] may be crucial to minimize MOVs in children aged 12–23 months in SSA.

More interestingly, this is the first study to assess factors associated with uncorrected, all corrected, and some corrected MOVs in SSA. The strength of this status is to review factors driving MOVs indicators in SSA. Compared to previous studies that only focus on MOVs-related barriers, this study is the first to consider the barriers associated with the most important MOVs indicators. Another strength of this research was its ability to categorize obstacles in caregivers and health systems, as well as perform robust multilevel logistic and multinomial logistic regression analyses. In addition, this study included multiple limitations, related to missing data. Nevertheless, only the statistically significance of father education changed to no significance after running multiple imputation for missing data. MOVs barriers related to health workers were not reported in the surveys. The sensitivity analysis including random-effects vs. fixed-effects conditional logistic models showed that our results were not robust enough. This could be explained by the fact that unobserved differences between countries are substantial enough that the “averaging” effect of the random-effects model obscures the specific local relationships captured by the fixed-effects model. Only the survey conducted in Nigeria [28] included MOVs barriers related to health workers and vaccine hesitancy, such as long waiting time, vaccine stock out, staff shortage, and health workers’ attitude, knowledge, and capability to assess children’s immunisation status and needs, beliefs about immunisation, fear of vaccine-preventable diseases, and immunisation safety concerns. However, these variables were excluded from the study due to their lack of significance and the inaccuracies in their data collection.

## 5. Conclusions

This study has demonstrated that multiple factors are driving MOVs indicators in children aged 12–23 months in SSA, including crude MOVs, all corrected, uncorrected, and some corrected MOVs. These MOV indicators were associated with mothers’ and fathers’ educational levels, religion, households with health insurance, mode of transportation, and occupation. This study also highlights that mothers’ education levels, health-insured children, and delivery places constitute a major factor influencing MOVs indicators in children aged 12–23 months in SSA. In addition, limited media access may either increase or decrease MOVs indicators. Multicomponent or multilevel strategies, such as radio-based health promotion campaigns targeting MOVs hot spot areas, educational interventions, expanding insurance coverage, IMCI, may be a useful strategy for raising community awareness and encouraging mothers to vaccinate their children in SSA. Effective multicomponent or multilevel strategies targeting MOVs barriers, including caregivers, health workers, and health system, are needed to reduce MOVs in children aged 12–23 months in SSA in view of reaching the immunization Agenda 2030 and beyond.

## Figures and Tables

**Table 1:** Sample size in each country, and total sample size in sub-Saharan Africa.

Number	SSA Region	Country	Survey	Year	Total Weighted Frequency	Percentage (%)
1	Western	Benin	MICS	2021–2022	1828	7.78
2	Western	Burkina Faso	DHS	2021	1035	4.41
3	Central	Chad	MICS	2019	1154	4.91
4	Eastern	Comoros	MICS	2021	652	2.77
5	Western	Cote d’Ivoire	DHS	2021	806	3.43
6	Southern	Eswatini	MICS	2021–2022	367	1.56
7	Central	Gabon	DHS	2019–2021	780	3.32
8	Western	Gambia	DHS	2019–2020	759	3.23
9	Western	Ghana	DHS	2022	899	3.83
10	Eastern	Kenya	DHS	2022	2608	11.10
11	Western	Liberia	DHS	2019–2020	338	1.44
12	Southern	Madagascar	DHS	2021	658	2.80
13	Southern	Malawi	MICS	2020	2519	10.72
14	Southern	Mozambique	DHS	2022–2023	564	2.40
15	Western	Nigeria	MICS	2021	3083	13.12
16	Eastern	Rwanda	DHS	2019–2020	753	3.20
17	Central	São Tomé and Principe	MICS	2019	317	1.35
18	Western	Sierra Leonne	DHS	2019	731	3.11
19	Western	Senegal	DHS	2019 and 2023	1789	7.62
20	Eastern	Tanzania	DHS	2022	852	3.63
21	Southern	Zimbabwe	MICS	2019	998	4.25

**Table 2:** Multilevel fixed-effects logistic regression analysis in assessing factors associated with MOVs among children aged 12–23 months in SSA.

Variables	Bivariable Analysis		Multivariable Analysis	
	Crude OR (95%CI)	*p*-Value	Adjusted OR (95%CI)	*p*-Value
**Caregiver’s Variables**				
**Women aged 15–49 years**				
15–24	1		1	
25–34	0.92 (0.84–1.01)	0.068	0.85 (0.53–1.35)	0.487
35–49	0.91 (0.82–1.03)	0.135	0.68 (0.39–1.17)	0.165
**Child’s gender**				
Boy	1			
Girl	0.98 (0.92–1.06)	0.665	-	-
**Number of children under five**				
One	1		1	
2–3	1.20 (1.11–1.29)	<0.001 ***	1.09 (0.68–1.75)	0.729
≥4	1.26 (1.07–1.47)	0.004 **	1.04 (0.55–1.98)	0.900
**Age of the head of household**				
15–24	1			
25–34	1.07 (0.92–1.26)	0.366	-	-
35–44	0.99 (0.84–1.15)	0.867	-	-
45–54	1.12 (0.94–1.33)	0.192	-	-
≥55	0.96 (0.81–1.13)	0.614	-	-
**Sex of household head**				
Male	1			
Female	1.02 (0.93–1.12)	0.654	-	-
**Household members**				
1–3	1		1	
4–5	1.02 (0.91–1.15)	0.662	0.98 (0.43–2.23)	0.973
≥6	1.16 (1.04–1.30)	0.010 *	0.73 (0.33–1.61)	0.439
**Mode of transportation to the nearest health facility**				
walking	1		1	
car/truck/bus/taxi/boat motor	0.85 (0.72–1.00)	0.058	0.85 (0.53–1.38)	0.521
bicycle/animal-drawn/boat no motor	0.76 (0.62–0.93)	0.009 **	0.41 (0.24–0.70)	0.001 **
**Caretaker’s education**				
No education	1		1	
Primary	0.84 (0.77–0.91)	<0.001 ***	0.54 (0.31–0.92)	0.025 *
Secondary or higher	0.75 (0.68–0.83)	<0.001 ***	0.46 (0.25–0.84)	0.012 *
**Father’s education**				
no education	1		1	
primary	0.79 (0.69–0.91)	0.001 **	2.34 (1.30–4.21)	0.005 **
secondary or higher	0.88 (0.76–1.03)	0.107	1.13 (0.63–2.03)	0.685
unknown	1.15 (0.91–1.45)	0.246	1.66 (0.68–2.38)	0.267
**Residence**				
Urban	1		1	
Rural	1.48 (1.11–2.08)	0.003 **	1.14 (0.98–1.65)	0.326
**Occupation**				
Not working	1		1	
Manual/HH/Domestic	0.82 (0.69–0.97)	0.021 *	1.20 (0.60–2.38)	0.609
Sales	0.51 (0.45–0.58)	<0.001 ***	0.50 (0.30–0.83)	0.008 **
Agriculture	0.61 (0.51–0.73)	<0.001 ***	0.58 (0.17–1.92)	0.374
Pro/Tec/Man/Cler/Ser/Other	0.81 (0.69–0.94)	0.008 **	0.82 (0.46–1.46)	0.496
**Wealth index quintile**				
Poorest	1		1	
Second	0.90 (0.81–1.01)	0.068	1.24 (0.73–2.09)	0.421
Middle	0.88 (0.79–0.98)	0.025 *	0.76 (0.42–1.38)	0.373
Fourth	0.85 (0.76–0.96)	0.009 **	1.16 (0.59–2.26)	0.668
Richest	0.79 (0.70–0.89)	<0.001 ***	0.72 (0.31–1.67)	0.441
**Religion**				
Animist/Traditionalist/No religion	1		1	
Muslim	0.70 (0.60–0.81)	<0.001 ***	1.34 (0.51–3.53)	0.553
Catholic	1.17 (0.98–1.38)	0.073	2.13 (0.72–6.32)	0.170
Protestant	0.58 (0.49–0.69)	<0.001 ***	0.76 (0.19–3.12)	0.707
Other Christian	0.72 (0.63–0.83)	<0.001 ***	1.56 (0.57–4.21)	0.383
**Listening to the radio**				
No	1		1	
Yes	0.64 (0.59–0.68)	<0.001 ***	0.49 (0.33–0.73)	<0.001 ***
**Watching TV**				
No	1		1	
Yes	0.88 (0.80–0.97)	0.010 *	0.97 (0.61–1.53)	0.886
**Internet**				
No	1		1	
Yes	0.83 (0.76–0.91)	<0.001 ***	0.83 (0.48–1.40)	0.481
**Reading newspaper or magazine**				
No	1			
Yes	1.04 (0.87–1.23)	0.656	-	-
**Health system’s variables**				
**Place of delivery**				
Home	1		1	
Public	0.69 (0.60–0.79)	<0.001 ***	0.37 (0.21–0.67)	0.001 **
Private	0.65 (0.49–0.86)	0.002 **	1.26 (0.28–5.60)	0.764
NGO/Religious	0.62 (0.41–0.95)	0.030 *	1	-
Other	0.85 (0.50–1.45)	0.552	0.12 (0.01–1.09)	0.059
**Place of vaccination**				
Outreach/Campaign	1		1	
Public	1.14 (0.86–1.51)	0.357	0.84 (0.41–1.73)	0.640
Private	0.67 (0.40–1.15)	0.146	0.60 (0.10–3.62)	0.575
NGO/Religious	1.93 (0.99–3.78)	0.054	1	-
**Health insurance**				
No	1		1	
Yes	0.96 (0.81–1.13)	0.130	0.44 (0.21–0.89)	0.023 *
**Visited health facility the last 12 months**				
No	1		1	
Yes	0.73 (0.64–0.83)	<0.001 ***	0.98 (0.65–1.47)	0.936
**Reason not using health facility/lack of access/too far**				
No	1		1	
Yes	0.67 (0.17–2.64)	0.563	-	-
**Reason not using health facility/costs too much**				
No	1		1	
Yes	7.22 (3.39–15.37)	<0.001 ***	3.34 (0.71–15.74)	0.127
**Distance to health facility**				
Not a big problem	1		1	
Big problem	1.47 (1.31–1.64)	<0.001 ***	1.31 (0.84–2.05)	0.228
**Time to the nearest health facility**				
Less than one hour	1		1	
One hour	1.19 (1.03–1.37)	0.017 *	1.43 (0.92–2.22)	0.111
Two hours	1.70 (1.35–2.15)	<0.001 ***	1.27 (0.59–2.72)	0.539
Three hours	1.50 (1.00–2.27)	0.051	1.62 (0.48–5.43)	0.432
More than three hours	1.46 (0.85–2.51)	0.173	0.38 (0.09–1.59)	0.187

Note *** = *p* < 0.001, ** = *p* < 0.01, * = *p* < 0.05.

**Table 3:** Multilevel fixed-effects multinomial logistic regression analysis assessing factors associated with corrected MOVs status among children aged 12–23 months in SSA.

Variables	All Corrected MOVs		Some Corrected MOVs	
	ARRR (95%CI)	*p*-Values	ARRR (95%CI)	*p*-Values
**Caregiver’s Variables**				
**Number of under five**				
One	1		1	
2–3	1.05 (0.75–1.46)	0.793	0.96 (0.63–1.47)	0.858
≥4	1.17 (0.73–1.85)	0.512	0.86 (0.46–1.61)	0.635
**Mother’s education**				
No education	1		1	
Primary	1.80 (1.22–2.65)	0.003 **	1.65 (1.02–2.65)	0.040 *
Secondary or higher	1.71 (1.08–2.70)	0.022 *	0.65 (0.35–1.21)	0.172
**Father’s education**				
No education	1		1	
Primary	1.23 (0.81–1.84)	0.327	1.60 (0.94–2.72)	0.083
Secondary or higher	1.31 (0.83–2.07)	0.238	1.08 (0.61–1.92)	0.779
Unknown	1.45 (0.70–3.03)	0.316	2.04 (0.84–5.00)	0.117
**Religion**				
Animist/Traditionalist/No religion	1		1	
Muslim	2.03 (1.05–3.91)	0.034 *	1.40 (0.66–2.98)	0.379
Catholic	1.59 (0.76–3.33)	0.218	1.66 (0.72–3.81)	0.231
Protestant	0.72 (0.26–2.00)	0.531	1.07 (0.34–3.38)	0.911
Other Christian	1.66 (0.85–3.25)	0.135	0.70 (0.30–1.61)	0.399
**Residence**				
Rural	1		1	
Urban	0.69 (0.47–1.00)	0.053	1.22 (0.77–1.91)	0.391
**Listening to the Radio**				
No	1		1	
Yes	1.50 (1.11–2.03)	0.009 **	0.99 (0.68–1.45)	0.968
**Watching TV**				
No	1		1	
Yes	0.61 (0.43–0.87)	0.006 **	0.61 (0.40–0.93)	0.022 *
**Internet**				
No	1		1	
Yes	1.34 (0.90–2.00)	0.147	0.98 (0.56–1.72)	0.952
**Reading newspaper or magazine**				
No	1		1	
Yes	1.47 (0.75–2.85)	0.257	1.45 (0.57–3.67)	0.433
**Health system ‘variables**				
**Distance to health facility**				
Big problem	1		1	
Not a big problem	0.87 (0.63–1.19)	0.390	0.74 (0.50–1.11)	0.146
**Vaccination place**				
Outreach/Campaign	1		1	
Public	0.89 (0.51–1.52)	0.664	1.16 (0.55–2.46)	0.684
Private	0.79 (0.16–0.40)	0.776	1.42 (0.19–10.69)	0.730
NGO/Religious	0.68 (0.11–4.22)	0.681	0.00 (0.00–0.00)	<0.001 ***
**Place of delivery**				
Home	1		1	
Public	1.07 (0.73–1.58)	0.719	0.64 (0.40–1.02)	0.063
Private	0.36 (0.09–1.43)	0.146	0.04 (0.01–0.22)	<0.001 ***
NGO/Religious	3.69 (0.16–11.41)	0.416	8.90 (0.64–11.65)	0.084
Other	1.32 (0.30–5.71)	0.710	2.93 (0.48–17.86)	0.244
**Health insurance**				
No	1		1	
Yes	2.53 (1.54–4.14)	<0.001 ***	2.15 (1.17–3.95)	0.013 *

Note *** = *p* < 0.001, ** = *p* < 0.01, * = *p* < 0.05.

**Table 4:** Model fitness of factors associated with MOVs indicators in SSA.

**Multilevel Random-Effects Logistic Regression Analysis**				
**Parameters**	**Null Model**	**Model 1**	**Model 2**	**Model 3**
LLR	−13,737.86	−2685.45	−740.37	−514.58
AIC	27,479.72	5432.91	1516.74	1117.16
BIC	27,495.84	5633.74	1609.65	1332.92
**Multilevel random-effects multinomial logistic regression analysis**				
LLR	−8138.60	−2671.21	−2074.75	−1340.88
AIC	16,283.2	5410.43	4189.50	2785.77
BIC	16,304.03	5615.29	4302.06	3060.22

Note: AIC: Akaike’s Information Criterion, BIC: Bayesian Information Criterion, LLR: Log-likelihood Ratio.

**Table 5:** Random-effects model assessing factors associated with MOVs indicators in SSA.

**(a): Multilevel Random-Effects Logistic Regression Analysis**				
**Parameters**	**Null Model**	**Model 1**	**Model 2**	**Model 3**
Community-level variance	0.51	0.13	0.30	0.12
ICC	0.32	0.21	0.22	0.18
PCV	Ref	0.74	0.41	0.76
MOR (95%CI)	4.91 (2.81–5.98)	1.29 (0.68–1.89)	1.37 (0.20–2.54)	2.37 (1.29–3.45)
**(b): Multilevel random-effects multinomial logistic regression analysis**				
Community-level variance	0.49	0.19	0.26	0.17
ICC	0.43	0.27	0.19	0.27
PCV	Ref	0.61	0.47	0.65
MOR (95%CI)	4.71 (2.75–5.84)	2.59 (2.13–3.06)	2.99 (2.36–3.62)	2.58 (2.23–4.13)

Note: MOR: Median Odds Ratio, PCV: proportional change in variance.

## Data Availability

All data used in this study can be downloaded from the following organizations: USAID DHS project (http://dhsprogram.com/data/available-datasets.cfm, accessed on 12 October 2024 ), UNICEF MICS project (http://mics.unicef.org/surveys, accessed on 24 November 2024). The data presented in this study are available on request from the corresponding author due to ethical reasons.

## Supplementary Materials

The following supporting information can be downloaded at: https://www.mdpi.com/article/10.3390/vaccines14050417/s1, Figure S1: Model 1 using Generalized Structural Equation Modelling (GSEM) for multilevel multinomial logistic regression analysis; Figure S2: Model 2 using Generalized Structural Equation Modelling (GSEM) for multilevel multinomial logistic regression analysis; Figure S3: Model 3 using Generalized Structural Equation Modelling (GSEM) for multilevel multinomial logistic regression analysis; Table S1. Definitions and categories of explanatory variables; Table S2: Multicollinearity between explanatory variables using the Variance Inflation Factor (VIF); Table S3: Description of factors driving MOVs among children aged 12–23 months in SSA; Table S4: Description of potential factors driving uncorrected, all corrected and some corrected MOVs among children aged 12–23 months in SSA; Table S5: Comparison of estimates of the multilevel fixed-effects logistic regression analysis before and after imputation in assessing factors associated with MOVs among children aged 12–23 months in SSA; Table S6: Sensitivity analysis checking the robustness of random-effects vs fixed-effects conditional logistic models in assessing factors associated with MOVs among children aged 12–23 months in SSA; Table S7: Bivariable multinomial logistic regression analysis assessing factors associated with MOVs correction status among children aged 12–23 months in sub-Saharan Africa; Table S8: Null Model, Model 1, and 2 of multilevel logistic regression analysis in assessing factors associated with MOVs among children aged 12–23 months in SSA; Table S9: Predicted posterior mean of MOVs in children aged 12–23 months using Model 3 in SSA; Table S10: Model-predicted probability of uncorrected, corrected, and some corrected MOVs in multinomial logistic regression analysis using key variables.

## Abbreviations

BCG: Bacillus Calmette-Guerin, COVID-19: Coronavirus disease, DHS: Demographic and Health Survey, DTP: diphtheria, tetanus and pertussis, EPI: Expanded Programme on Immunization, FOCUS: (F = Find a problem, O = Organize a team, C = Clarify the problem, U = Understand a problem, S = Select an intervention), FVL: forms and variable Lists, GSEM, Generalized Structural Equation Modelling, HepB: hepatitis B, Hib: Haemophilus influenzae type b, ICC: intraclass correlation coefficient, MCV: measles-containing vaccine, LLR: log-likelihood ratio, MICS: Multiple Indicator Cluster Surveys, MOV: missed opportunities for vaccination, PCV: pneumococcal conjugate vaccine, PDSA (P = Plan, D = Do, S = Study, A = Act), PSUs: Primary sampling units, RI: Routine Immunization, SSA: Sub-Saharan Africa, TV: television, UNICEF: United Nations International Children’s Emergency Fund, USAID: United States Agency for International Development, VCQI: vaccination coverage quality indicators, VIF: variance inflation factor, WHO: World Health Organization.
