# Supplementary material for: Determinants of Missed Opportunities for Vaccination (MOVs) Indicators Among Children Aged 12–23 Months in Sub-Saharan African Countries: A Multilevel Analysis of Survey Data
Source: Vaccines (Basel). 2026 May 6;14(5):417. doi: 10.3390/vaccines14050417 (PMC13211415; doi:10.3390/vaccines14050417)
Supplement: Supplementary file 1 [file vaccines-14-00417-s001.zip › vaccines-4019060-supplementary.pdf]

Supplemental materials: Determinant factors driving missed opportunities for vaccination (MOVs) indicators among children aged 12–23 months in sub-Saharan African countries: A multilevel analysis from DHS and MICS data surveys.

Supplemental Table S1. Definitions and categories of explanatory variables.

Variables	Definitions and categories
Gender of Child	Categorised as boy = 1 or girl = 2
Age of mother	Maternal age during childbirth categorised in 15-24, 25-34, and 35-49 years
Age of household head	Categorized as 15-24, 25-34, 35-44, 45-54, and ≥55
Household numbers	Categorized by the number of people in the household: fewer than or equal to three, four to five, and six or more people.
Residence	Type of place of residence categorised as follows: 1 = rural and 2 = urban.
Number of children under five	Categorized by the number of children under the age of five in the household: one, two to three, and equal to or more than four children.
Sex of household head	Sex of household head (0 = male and 1 = female)
Mothers' education	Mothers' education levels were classified as 0 (no formal education), 1 (primary), and 2 (secondary and higher).
Fathers' education	Categorized in no formal education, primary, secondary or higher, and unknown.
Occupation	Working status of the head of household classified as follows: not working, manual/HH/domestic, agriculture, sales, and Pro/Tec/Man/Cler/Ser.
Religion	Categorized as Animist/Traditionalist/No religion, Muslim, Catholic, Protestant, and other Christians.
Wealth index quintile	The categories were the poorest (=1), second (=2), middle (=3), fourth (=4), and wealthiest (=5).
Means of transportation	Mean of transportation to go to the health facility, which included Walking, biking, motorcycling, driving, using the bus, and other ways
Household exposure to media.	Household media exposure (0 = no exposure to newspapers, radio, internet, or television; 1 = exposure to one or more of these).

Visited health facility the last twelve months	Women who were not using birth control and were visited by a fieldworker who talked about family planning, went to a health facility and talked about family planning, went to a health facility but did not talk about family planning, or did not talk about family planning with a fieldworker or at a health facility in the 12 months before the survey
Health insurance	Grouped into two groups: children with health insurance (=1) and children without health insurance (=0)
Vaccination place	Defined as the venue where the child was largely vaccinated, including outreach/campaigns, public, private, and NGO/Religious health institutions.
Reason not using health facility/costs too much	Categorised as not costly = 1 or costly = 2.
Reason not using health facility/lack of access/too far	Categorised as “No” = 1 or “Yes” = 2.
Place of delivery	Categories included public/government service, private, non-profit, faith-based organizations, and home.
Distance to health facility	Perceived distance to nearest health facility (0 = not a big, 1 = a big problem).
Time to the nearest facility	Grouped into four groups: Less than one hour, One hour, Two hours, Three hours, and more than more than three hours

Supplemental Table S2. Multicollinearity between explanatory variables using the Variance Inflation Factor (VIF)

Number	**Variable**	**VIF**	**1/VIF**
1	**Gender**	1.02	0.983
2	**Number of children under 5**	1.32	0.758
3	**Age of the head of household**	1.38	0.723
4	**Wealth index**	1.89	0.528

5	**Number of household member**	1.61	0.621
6	**Residence**	1.57	0.636
7	**Mothers' education**	1.63	0.612
8	**Religion**	1.53	0.654
9	**Watching TV**	1.47	0.681
10	**Health insurance**	1.47	0.682
11	**Sex of household head**	1.06	0.946
12	**internet**	1.37	0.732
13	**Distance to the nearest health facility**	1.32	0.755
14	**Time to the nearest health facility**	1.25	0.800
15	**Father's education**	1.44	0.696
16	**Reading newspaper**	1.17	0.856
17	**Listening to the radio**	1.14	0.877
18	**Place of delivery**	1.13	0.881
19	**Place of vaccination**	1.08	0.924
20	**Visited health facility last 12 months**	1.05	0.952
21	**Occupation**	1.04	0.959
22	**Reason not using: lack of access/too far**	1.03	0.970
23	**Reason not using: costs too much**	1.03	0.975
24	**Mode of transportation**	1.13	0.884
Mean VIF		1.30	

Supplemental Table S3. Description of factors driving MOVs among children aged 12-23 months in SSA

Variables	Total	No MOV	MOV	p-value
	N=23,490	N=15,841	N=7,649	
**Caregiver's variables**				
**Women aged 15-49 years**				<0.001***
15-24	13,015 (55.43%)	8,612 (54.39%)	4,403 (57.56%)	
25-34	7,469 (31.81%)	5,195 (32.81%)	2,274 (29.73%)	
35-49	2,998 (12.77%)	2,026 (12.80%)	972 (12.71%)	
**Child's gender**				0.29
Male	11,999 (51.08%)	8,130 (51.32%)	3,869 (50.58%)	
Female	11,491 (48.92%)	7,711 (48.68%)	3,780 (49.42%)	
**Number of children under five**				<0.001***
One	10,033 (42.73%)	7,020 (44.34%)	3,013 (39.39%)	
2-3	11,436 (48.70%)	7,489 (47.30%)	3,947 (51.60%)	
≥4	2,013 (8.57%)	1,324 (8.36%)	689 (9.01%)	
**Age of household head**				0.11
15-24	1,407 (5.99%)	947 (5.98%)	460 (6.01%)	
25-34	7,203 (30.67%)	4,857 (30.68%)	2,346 (30.67%)	
35-44	7,110 (30.28%)	4,845 (30.60%)	2,265 (29.61%)	
45-54	3,692 (15.72%)	2,422 (15.30%)	1,270 (16.60%)	
≥55	4,070 (17.33%)	2,762 (17.44%)	1,308 (17.10%)	
**Sex of household head**				0.20
Male	18,452 (78.58%)	12,479 (78.82%)	5,973 (78.09%)	
Female	5,030 (21.42%)	3,354 (21.18%)	1,676 (21.91%)	
**Household members**				<0.001***
≤3	3,172 (13.51%)	2,224 (14.05%)	948 (12.39%)	
4-5	7,248 (30.87%)	4,992 (31.53%)	2,256 (29.49%)	
≥6	13,062 (55.63%)	8,617 (54.42%)	4,445 (58.11%)	
**Mode of transportation to the nearest health facility**				0.010*
Walking	3,873 (60.91%)	2,372 (59.57%)	1,501 (63.15%)	
Car/truck/bus/taxi/boat motor	1,644 (25.85%)	1,052 (26.42%)	592 (24.91%)	
Bicycle/animal-drawn/boat with no motor	842 (13.24%)	558 (14.01%)	284 (11.95%)	
**Mother's education**				<0.001***
No education	7,894 (33.61%)	5,075 (32.04%)	2,819 (36.85%)	
Primary	7,355 (31.31%)	4,996 (31.54%)	2,359 (30.84%)	
Secondary or higher	8,241 (35.08%)	5,770 (36.42%)	2,471 (32.30%)	
**Father's education**				<0.001***
No education	4,187 (36.53%)	2,803 (35.65%)	1,384 (38.44%)	
Primary	2,837 (24.75%)	1,997 (25.40%)	840 (23.33%)	

Secondary or higher	3,803 (33.18%)	2,676 (34.03%)	1,127 (31.31%)	
Unknown	636 (5.55%)	387 (4.92%)	249 (6.92%)	
**Residence**				0.003**
Urban	7,556 (32.17%)	5,098 (32.18%)	2,458 (32.13%)	
Rural	15,934 (67.83%)	10,743 (67.82%)	5,191 (67.87%)	
**Occupation**				<0.001***
Not working	4,796 (37.96%)	2,965 (34.87%)	1,831 (44.33%)	
Manual/HH/Domestic	1,477 (11.69%)	986 (11.60%)	491 (11.89%)	
Sales	3,187 (25.23%)	2,366 (27.83%)	821 (19.88%)	
Pro/Tec/Man/Cler/Ser/Other	1,837 (14.54%)	1,198 (14.09%)	639 (15.47%)	
Agriculture	1,336 (10.58%)	988 (11.62%)	348 (8.43%)	
**Wealth index quintile**				<0.001***
Poorest	6,072 (25.85%)	3,901 (24.63%)	2,171 (28.38%)	
Second	5,209 (22.18%)	3,506 (22.13%)	1,703 (22.26%)	
Middle	4,680 (19.92%)	3,176 (20.05%)	1,504 (19.66%)	
Fourth	4,189 (17.83%)	2,910 (18.37%)	1,279 (16.72%)	
Richest	3,340 (14.22%)	2,348 (14.82%)	992 (12.97%)	
**Religion**				<0.001***
Animist/Traditionalist/No religion	1,873 (8.52%)	1,147 (7.69%)	726 (10.29%)	
Muslim	7,807 (35.52%)	5,387 (36.09%)	2,420 (34.31%)	
Catholic	2,224 (10.12%)	1,337 (8.96%)	887 (12.58%)	
Protestant	2,288 (10.41%)	1,706 (11.43%)	582 (8.25%)	
Other Christian	7,786 (35.43%)	5,348 (35.83%)	2,438 (34.57%)	
**Listening to the radio**				<0.001***
No	11,624 (50.18%)	7,302 (46.93%)	4,322 (56.84%)	
Yes	11,541 (49.82%)	8,259 (53.07%)	3,282 (43.16%)	
**Watching TV**				0.041*
No	9,923 (51.24%)	6,715 (50.74%)	3,208 (52.32%)	
Yes	9,444 (48.76%)	6,520 (49.26%)	2,924 (47.68%)	
**Internet**				<0.001***
No	16,810 (72.57%)	11,184 (71.87%)	5,626 (73.99%)	
Yes	6,355 (27.43%)	4,377 (28.13%)	1,978 (26.01%)	
**Reading newspaper or magazine**				0.76
No	11,197 (88.55%)	7,534 (88.49%)	3,663 (88.67%)	
Yes	1,448 (11.45%)	980 (11.51%)	468 (11.33%)	
**Health system's variables**				
**Place of delivery**				<0.001***
Other	93 (0.74%)	60 (0.70%)	33 (0.80%)	
Public	9,585 (75.81%)	6,582 (77.32%)	3,003 (72.69%)	
Private	754 (5.96%)	531 (6.24%)	223 (5.40%)	
NGO/Religious	235 (1.86%)	168 (1.97%)	67 (1.62%)	

Home	1,977 (15.64%)	1,172 (13.77%)	805 (19.49%)	
**Place of vaccination**				<0.001
Outreach/Campaign	1,275 (11.97%)	976 (13.04%)	299 (9.43%)	
Public	9,041 (84.85%)	6,258 (83.61%)	2,783 (87.79%)	
Private	280 (2.63%)	217 (2.90%)	63 (1.99%)	
NGO/Religious	59 (0.55%)	34 (0.45%)	25 (0.79%)	
**Health insurance**				0.48
No	16,621 (87.98%)	11,101 (87.87%)	5,520 (88.22%)	
Yes	2,270 (12.02%)	1,533 (12.13%)	737 (11.78%)	
**Visited health facility the last 12 months**				<0.001***
No	2,918 (25.61%)	1,786 (23.65%)	1,132 (29.46%)	
Yes	8,475 (74.39%)	5,765 (76.35%)	2,710 (70.54%)	
**Reason not using health facility/lack of access/too far**				0.21
No	2,718 (99.23%)	1,714 (99.08%)	1,004 (99.50%)	
Yes	21 (0.77%)	16 (0.92%)	5 (0.50%)	
**Reason not using health facility/costs too much**				<0.001***
No	2,673 (97.59%)	1,712 (98.96%)	961 (95.24%)	
Yes	66 (2.41%)	18 (1.04%)	48 (4.76%)	
**Distance**				<0.001***
Not a big problem	7,094 (63.88%)	5,013 (66.45%)	2,081 (58.44%)	
Big problem	4,011 (36.12%)	2,531 (33.55%)	1,480 (41.56%)	
**Time to the nearest facility**				<0.001***
Less than one hour	3,181 (50.02%)	2,087 (52.41%)	1,094 (46.02%)	
One hour	2,402 (37.77%)	1,466 (36.82%)	936 (39.38%)	
Two hours	517 (8.13%)	286 (7.18%)	231 (9.72%)	
Three hours	168 (2.64%)	88 (2.21%)	80 (3.37%)	
More than three hours	91 (1.43%)	55 (1.38%)	36 (1.51%)	

Note *** = p<0.001, ** = p<0.01, * = p<0.05

Supplemental Table S4. Description of potential factors driving uncorrected, all corrected and some corrected MOVs among children aged 12-23 months in SSA.

**Variables**	Total	Uncorrected MOVs	Corrected MOVs	Some corrected MOVs	p-value
	N=7,649	N=3,227	N=3,301	N=1,121	
**Caregiver's variables**					
**Number of children under five**					0.020*
One	3,013 (39.39%)	1,213 (37.59%)	1,362 (41.26%)	438 (39.07%)	
2-3	3,947 (51.60%)	1,714 (53.11%)	1,639 (49.65%)	594 (52.99%)	
≥4	689 (9.01%)	300 (9.30%)	300 (9.09%)	89 (7.94%)	

**Mother's education**					<0.001***
No education	2,819 (36.85%)	1,243 (38.52%)	1,129 (34.20%)	447 (39.88%)	
Primary	2,359 (30.84%)	873 (27.05%)	1,133 (34.32%)	353 (31.49%)	
Secondary or higher	2,471 (32.30%)	1,111 (34.43%)	1,039 (31.48%)	321 (28.64%)	
**Father's education**					<0.001***
no education	1,384 (38.44%)	550 (37.16%)	620 (39.02%)	214 (40.30%)	
primary	840 (23.33%)	279 (18.85%)	420 (26.43%)	141 (26.55%)	
secondary or higher	1,127 (31.31%)	509 (34.39%)	481 (30.27%)	137 (25.80%)	
Unknown	249 (6.92%)	142 (9.59%)	68 (4.28%)	39 (7.34%)	
**Religion**					<0.001
Animist/Traditionalist/No religion	726 (10.29%)	380 (12.49%)	238 (7.90%)	108 (10.80%)	
Muslim	2,420 (34.31%)	977 (32.12%)	1,086 (36.07%)	357 (35.70%)	
Catholic	887 (12.58%)	466 (15.32%)	277 (9.20%)	144 (14.40%)	
Protestant	582 (8.25%)	247 (8.12%)	266 (8.83%)	69 (6.90%)	
Other Christian	2,438 (34.57%)	972 (31.95%)	1,144 (37.99%)	322 (32.20%)	
**Residence**					<0.001***
Urban	2,458 (32.13%)	1,235 (38.27%)	886 (26.84%)	337 (30.06%)	
Rural	5,191 (67.87%)	1,992 (61.73%)	2,415 (73.16%)	784 (69.94%)	
**Listening to the radio**					<0.001***
No	4,322 (56.84%)	1,934 (60.23%)	1,709 (52.17%)	679 (60.79%)	
Yes	3,282 (43.16%)	1,277 (39.77%)	1,567 (47.83%)	438 (39.21%)	
**Watching TV**					<0.001***
No	3,208 (52.32%)	1,180 (46.22%)	1,526 (56.35%)	502 (57.63%)	
Yes	2,924 (47.68%)	1,373 (53.78%)	1,182 (43.65%)	369 (42.37%)	
**Internet**					<0.001***
No	5,626 (73.99%)	2,249 (70.04%)	2,524 (77.05%)	853 (76.37%)	
Yes	1,978 (26.01%)	962 (29.96%)	752 (22.95%)	264 (23.63%)	
**Reading newspaper or magazine**					0.068
No	3,663 (88.67%)	1,600 (87.86%)	1,525 (88.61%)	538 (91.34%)	
Yes	468 (11.33%)	221 (12.14%)	196 (11.39%)	51 (8.66%)	
**Health system's variables**					
**Distance**					<0.001***
Not a big problem	2,081 (58.44%)	800 (53.02%)	984 (64.06%)	297 (57.56%)	
Big problem	1,480 (41.56%)	709 (46.98%)	552 (35.94%)	219 (42.44%)	
**Vaccination Place**					0.009**
Outreach/Campaign	299 (9.43%)	83 (7.82%)	184 (11.46%)	32 (6.36%)	
Public	2,783 (87.79%)	950 (89.45%)	1,378 (85.86%)	455 (90.46%)	
Private	63 (1.99%)	21 (1.98%)	30 (1.87%)	12 (2.39%)	
NGO/Religious	25 (0.79%)	8 (0.75%)	13 (0.81%)	4 (0.80%)	
**Place of delivery**					<0.001

Home	805 (19.49%)	308 (16.91%)	342 (19.87%)	155 (26.32%)	
Public	3,003 (72.69%)	1,364 (74.90%)	1,239 (71.99%)	400 (67.91%)	
Private	223 (5.40%)	117 (6.43%)	85 (4.94%)	21 (3.57%)	
NGO/Religious	67 (1.62%)	19 (1.04%)	39 (2.27%)	9 (1.53%)	
Other	33 (0.80%)	13 (0.71%)	16 (0.93%)	4 (0.68%)	
**Health insurance**					
No	5,520 (88.22%)	2,316 (84.74%)	2,344 (91.14%)	860 (90.34%)	<0.001
Yes	737 (11.78%)	417 (15.26%)	228 (8.86%)	92 (9.66%)	

Note *** = p<0.001, ** = p<0.01, * = p<0.05

Supplemental Table S5. Comparison of estimates of the multilevel fixed-effects logistic regression analysis before and after imputation in assessing factors associated with MOVs among children aged 12-23 months in SSA.

Variables	Before Multiple Imputation		After Multiple Imputation	
	Adjusted OR (95%CI)	P-value	Adjusted OR (95%CI)	P-value
**Caregiver's variables**				
**Women aged 15-49 years**				
15-24	1		1	
25-34	0.85(0.53 - 1.35)	0.487	1.12(0.93 - 1.33)	0.228
35-49	0.68(0.39 - 1.17)	0.165	0.88(0.70- 1.09)	0.246
**Number of children under five**				
One	1		1	
2-3	1.09(0.68 - 1.75)	0.729	1.19(0.99 - 1.43)	0.067
≥4	1.04(0.55 - 1.98)	0.900	0.94(0.72 - 1.24)	0.673
**Household members**				
1-3	1		1	
4-5	0.98(0.43 - 2.23)	0.973	1.28(0.97- 1.70)	0.077
≥6	0.73(0.33 - 1.61)	0.439	1.01(0.76 - 1.34)	0.934
**Mode of transportation to the nearest health facility**				
walking	1		1	
car/truck/bus/taxi/boat motor	0.85(0.53 - 1.38)	0.521	0.92(0.77 – 1.11)	0.385
bicycle/animal-drawn/boat no motor	0.41(0.24 – 0.70)	0.001**	0.49(0.39 - 0.61)	<0.001**
**Caretaker's education**				
No education	1		1	
Primary	0.54(0.31 – 0.92)	0.025*	0.84(0.66 - 0.98)	0.042*
Secondary or higher	0.46(0.25 – 0.84)	0.012*	0.78(0.62 - 0.97)	0.031*
**Father's education**				
no education	1		1	
primary	2.34(1.30 - 4.21)	0.005**	1.10(0.88 - 1.41)	0.417
secondary or higher	1.13(0.63 - 2.03)	0.685	1.12(0.88 - 1.44)	0.356
unknown	1.66(0.68 - 2.38)	0.267	0.93(0.65 - 1.34)	0.714
**Residence**				
Urban	1		1	1

Rural	1.14(0.98 -1.65)	0.326	1.22(0.92-1.70)	0.244
**Occupation**				
Not working	1		1	
Manual/HH/Domestic	1.20(0.60 - 2.38)	0.609	0.79(0.61 - 1.03)	0.084
Sales	0.50(0.30 – 0.83)	0.008**	0.54(0.43 - 0.66)	<0.001***
Agriculture	0.58(0.17 - 1.92)	0.374	0.50(0.33 – 0.78)	0.002**
Pro/Tec/Man/Cler/Ser/Other	0.82(0.46 - 1.46)	0.496	0.78(0.62 - 0.98)	0.035*
**Wealth index quintile**				
Poorest	1		1	
Second	1.24(0.73 - 2.09)	0.421	1.10(0.87 - 1.38)	0.419
Middle	0.76(0.42 - 1.38)	0.373	0.97(0.76 - 1.25)	0.833
Fourth	1.16(0.59 - 2.26)	0.668	1.00(0.76 - 1.32)	0.970
Richest	0.72(0.31 - 1.67)	0.441	0.96(0.70 - 1.32)	0.810
**Religion**				
Animist/Traditionalist/No religion	1		1	
Muslim	1.34(0.51 - 3.53)	0.553	0.89(0.60 - 1.31)	0.556
Catholic	2.13(0.72 - 6.32)	0.170	1.43(0.94 - 2.18)	0.095
Protestant	0.76(0.19 - 3.12)	0.707	0.79(0.47 - 1.34)	0.390
Other Christian	1.56(0.57 - 4.21)	0.383	1.19(0.80 - 1.32)	0.378
**Listening to the radio**				
No	1		1	
Yes	0.49(0.33 – 0.73)	<0.001***	0.72(0.62 – 0.85)	<0.001***
**Watching TV**				
No	1		1	
Yes	0.97(0.61 - 1.53)	0.886	0.91(0.74 – 1.10)	0.312
**Internet**				
No	1		1	
Yes	0.83(0.48 - 1.40)	0.481	0.83(0.68 - 1.01)	0.061
**Health system's variables**				
**Place of delivery**				
Home	1		1	
Public	0.37(0.21 – 0.67)	0.001**	0.79(0.24 - 1.15)	0.108
Private	1.26(0.28 – 5.60)	0.764	0.58(0.31 - 1.91)	0.581
NGO/Religious	1	-	1.76(0.26 - 11.98)	0.560
Other	0.12(0.01 - 1.09)	0.059	1.11(0.49 - 2.53)	0.788
**Place of vaccination**				
Outreach/Campaign	1		1	
Public	0.84(0.41 - 1.73)	0.640	0.79(0.59 - 1.07)	0.130
Private	0.60(0.10 - 3.62)	0.575	0.58(0.28 - 1.22)	0.152
NGO/Religious	1	-	0.79(0.18 - 3.48)	0.754
**Health insurance**				
No	1		1	
Yes	0.44(0.21 – 0.89)	0.023*	0.45(0.35 – 0.58)	<0.001***
**Visited health facility the last 12 months**				
No	1		1	
Yes	0.98(0.65 - 1.47)	0.936	0.92(0.78 - 1.09)	0.356
**Reason not using health facility/costs too much**				
No	1		1	
Yes	3.34(0.71 - 15.74)	0.127	1.25(0.56 - 2.79)	0.570
**Distance to health facility**				

Not a big problem	1		1	
Big problem	1.31(0.84 - 2.05)	0.228	1.00(0.84 - 1.19)	0.990
**Time to the nearest health facility**				
Less than one hour	1		1	
One hour	1.43(0.92 - 2.22)	0.111	1.35(1.14 - 1.61)	0.001**
Two hours	1.27(0.59 - 2.72)	0.539	1.55(1.14 - 2.11)	0.005**
Three hours	1.62(0.48 - 5.43)	0.432	1.79(0.79 - 1.30)	0.363
More than three hours	0.38(0.09 - 1.59)	0.187	1.07(0.60 - 1.89)	0.822

Note *** = p<0.001, ** = p<0.01, * = p<0.05.

Supplemental Table S6. Sensitivity analysis checking the robustness of random-effects vs fixed-effects conditional logistic models in assessing factors associated with MOVs among children aged 12-23 months in SSA.

**Variables**	**Random-Effects**		**Fixed-Effects**	
	Adjusted OR (95%CI)	P-value	Adjusted OR (95%CI)	P-value
**Caregiver's variables**				
**Women aged 15-49 years**				
15-24	1		1	
25-34	0.80(0.55 - 1.17)	0.253	0.78(0.55 - 1.11)	0.173
35-49	0.65(0.43 - 1.05)	0.083	0.78(0.53 - 1.16)	0.227
**Number of children under five**				
One	1		1	
2-3	1.15(0.78 - 1.70)	0.467	1.15(0.82 - 1.63)	0.413
≥4	1.16(0.69 - 1.97)	0.573	1.42(0.88 - 2.30)	0.152
**Household members**				
1-3	1		1	
4-5	0.82(0.42 - 1.60)	0.567	0.90(0.49 - 1.65)	0.741
≥6	0.71(0.37 - 1.36)	0.305	0.84(0.47 - 1.53)	0.578
**Mode of transportation to the nearest health facility**				
walking	1		1	
car/truck/bus/taxi/boat motor	0.83(0.57 - 1.24)	0.373	0.97(0.69 - 1.34)	0.839
bicycle/animal-drawn/boat no motor	0.66(0.40 - 1.08)	0.099	0.67(0.42 - 1.06)	0.088
**Caretaker's education**				
No education	1		1	
Primary	0.53(0.34 – 0.85)	0.009**	0.62(0.41 – 0.94)	0.025*
Secondary or higher	0.47(0.28 – 0.79)	0.004**	0.78(0.50 - 1.23)	0.284
**Father's education**				
no education	1		1	
primary	1.66(1.01 - 2.72)	0.044*	1.69(1.12 - 2.56)	0.013*
secondary or higher	0.98(0.57 - 1.49)	0.935	1.22(0.79 - 1.87)	0.372
unknown	1.49(0.65 - 2.82)	0.291	1.44(0.71 - 2.92)	0.314
**Residence**				
Urban	1		1	1
Rural	1.23(0.59 – 1.81)	0.227	1.33(0.51 – 1.67)	0.147

**Occupation**				
Not working	1		1	
Manual/HH/Domestic	1.51(0.85 - 2.69)	0.159	1.13(0.66 - 1.91)	0.658
Sales	0.63(0.41 – 0.97)	0.035*	0.82(0.56 - 1.21)	0.327
Agriculture	0.77(0.47 - 1.26)	0.303	0.74(0.51 - 1.20)	0.265
Pro/Tec/Man/Cler/Ser/Other	0.66(0.25 - 1.76)	0.409	0.78(0.30 - 1.84)	0.521
**Wealth index quintile**				
Poorest	1		1	
Second	1.12(0.74 - 1.73)	0.580	0.98(0.68 - 1.43)	0.934
Middle	0.75(0.45 - 1.23)	0.248	0.76(0.49 – 1.20)	0.243
Fourth	1.07(0.59 - 1.94)	0.815	1.27(0.74 - 2.18)	0.379
Richest	0.81(0.37 - 1.74)	0.583	0.78(0.34 - 1.53)	0.393
**Religion**				
Animist/Traditionalist/No religion	1		1	
Muslim	1.31(0.60 - 2.89)	0.495	1.24(0.63 - 2.43)	0.530
Catholic	1.69(0.70 - 4.01)	0.236	1.41(0.66 - 3.04)	0.373
Protestant	0.87(0.28 - 2.73)	0.812	0.76(0.26 - 2.22)	0.623
Other Christian	1.22(0.54 - 2.74)	0.630	0.76(0.39 - 1.51)	0.439
**Listening to the radio**				
No	1		1	
Yes	0.61(0.44 – 0.84)	0.003**	0.65(0.49 – 0.87)	0.004**
**Watching TV**				
No	1		1	
Yes	1.06(0.73 - 1.55)	0.751	1.01(0.72 - 1.42)	0.937
**Internet**				
No	1		1	
Yes	1.03(0.65 - 1.63)	0.909	0.82(0.54 - 1.26)	0.374
**Health system's variables**				
**Place of delivery**				
Home	1		1	
Public	0.66(0.43 – 0.89)	0.003**	0.55(0.33 – 0.81)	0.005**
Private	1.55(0.13 - 2.32)	0.414	1.64(0.41 - 6.54)	0.478
NGO/Religious	1	-	1	
Other	0.91(0.76 – 1.71)	0.081	1.01(0.78 – 1.93)	0.113
**Place of vaccination**				
Outreach/Campaign	1		1	
Public	0.77(0.42 – 1.41)	0.397	0.66(0.40 - 1.09)	0.106
Private	0.55(0.14 – 1.31)	0.414	0.70(0.15 - 3.23)	0.647
NGO/Religious	1		1	
**Health insurance**				
No	1		1	
Yes	0.64(0.31 - 1.33)	0.240	0.67(0.32 - 1.40)	0.285
**Visited health facility the last 12 months**				
No	1		1	
Yes	1.01(0.72 - 1.41)	0.962	0.91(0.98 - 1.23)	0.551
**Reason not using health facility/costs too much**				
No	1		1	
Yes	3.37(0.90 - 12.55)	0.070	1.92(0.62 – 6.00)	0.258
**Distance to health facility**				

Not a big problem	1		1	
Big problem	1.21(0.84 – 1.75)	0.284	1.16(0.84 - 1.59)	0.369
**Time to the nearest health facility**				
Less than one hour	1		1	
One hour	1.32(0.92 - 1.89)	0.132	1.16(0.84 - 1.59)	0.366
Two hours	1.09(0.57 - 2.07)	0.790	0.97(0.55 - 1.73)	0.929
Three hours	0.75(0.27 - 2.06)	0.575	1.01(0.41 - 2.47)	0.984
More than three hours	0.50(0.14 - 1.72)	0.273	0.45(0.13 - 1.52)	0.200

Note *** = p<0.001, ** = p<0.01, * = p<0.05.

Supplemental Table S7. Bivariable multinomial logistic regression analysis assessing factors associated with MOVs correction status among children aged 12-23 months in sub-Saharan Africa

**Variables**	**Uncorrected MOVs**	**All corrected MOVs**		**Some corrected MOVs**	
		Crude RRR (95%CI)	P-value	Crude RRR (95%CI)	P-value
**Women aged 15-49 years**					
15-24		1		1	
25-34		1.16(0.98 - 1.36)	0.081	0.86(0.69 - 1.07)	0.172
35-49		1.09(0.88 - 1.35)	0.441	0.92(0.69 - 1.23)	0.589
Child's gender					
Boy		1			
Girl		0.96(0.85 - 1.09)	0.541	1.01(0.85 – 1.20)	0.930
**Number of children under five**					
One		1		1	
2-3		0.82(0.71 – 0.94)	0.005**	0.99(0.83 - 1.19)	0.939
≥4		0.86(0.66 – 1.11)	0.240	0.89(0.65 - 1.23)	0.495
**Age of the head of household**					
15-24		1		1	
25-34		0.82(0.62 - 1.07)	0.146	0.96(0.66 - 1.38)	0.819
35-44		0.91(0.70 - 1.20)	0.518	0.94(0.65 - 1.36)	0.741
45-54		0.87(0.64 - 1.18)	0.377	0.96(0.64 - 1.44)	0.840
≥55		1.04(0.77 - 1.39)	0.811	0.85(0.57 - 1.26)	0.418
**Sex of household head**					
Male		1		1	
Female		1.12(0.95 - 1.32)	0.158	0.89(0.72 - 1.11)	0.303
**Household members**					
1-3		1		1	
4-5		0.86(0.70 - 1.06)	0.159	0.85(0.63 - 1.13)	0.266
≥6		0.92(0.75 - 1.14)	0.463	0.98(0.75 - 1.29)	0.903
**Mode of transportation to the nearest health facility**					
walking		1		1	
car/truck/bus/taxi/boat motor		1.16(0.88 - 1.54	0.295	0.67(0.47 – 0.95)	0.026*
bicycle/animal-drawn/boat no motor		1.34(0.95 - 1.89)	0.095	0.82(0.54 - 1.24)	0.354
**Mother's education**					

No education		1		1	
Primary		1.49(1.29 - 1.73)	<0.001***	1.06(0.86 – 1.30)	0.573
Secondary or higher		0.97(0.82 - 1.15)	0.755	0.69(0.55 – 0.86)	0.001**
**Father's education**					
no education		1		1	
primary		1.42(1.12 - 1.80)	0.004**	1.32(0.94 - 1.87)	0.107
secondary or higher		0.77(0.60 – 0.99)	0.042*	0.57(0.41 -0.80)	0.001**
unknown		0.56(0.37 – 0.85)	0.007**	0.88(0.53 - 1.45)	0.614
**Residence**					
Urban		1		1	
Rural		1.50(1.22 – 2.59)	<0.001***	1.57(1.17 – 1.70)	<0.001***
**Occupation**					
Not working		1			
Manual/HH/Domestic		1.12(0.84 - 1.48)	0.432	0.64(0.43 – 0.95)	0.029*
Sales		1.51(1.18 - 1.93)	0.001**	0.81(0.59 - 1.12)	0.199
Agriculture		1.22(0.88 - 1.69)	0.242	0.59(0.32 - 1.08)	0.090
Pro/Tec/Man/Cler/Ser/Other		0.94(0.72 - 1.24)	0.672	0.62(0.44 – 0.88)	0.007**
**Wealth index quintile**					
Poorest		1		1	
Second		0.84(0.70 - 1.02)	0.078	0.88(0.70 - 1.12)	0.317
Middle		0.85(0.70 - 1.03)	0.107	0.88(0.69 - 1.13)	0.328
Fourth		0.89(0.72 - 1.10)	0.291	0.89(0.68 - 1.16)	0.383
Richest		0.84(0.69 - 1.05)	0.129	0.75(0.55 - 1.01)	0.061
**Religion**					
Animist/Traditionalist/No religion		1		1	
Muslim		1.97(1.53 - 2.54)	<0.001***	1.40(1.02 - 1.92)	0.035*
Catholic		0.96(0.72 - 1.27)	0.778	1.25(0.86 - 1.82)	0.231
Protestant		1.90(1.39 - 2.59)	<0.001***	1.02(0.66 - 1.58)	0.910
Other Christian		1.97(1.53 - 2.53)	<0.001***	1.09(0.79 - 1.50)	0.609
**Listening to the radio**					
No		1		1	
Yes		1.39(1.21 - 1.58)	<0.001***	0.90(0.76 - 1.08)	0.258
**Television**					
No		1		1	
Yes		1.74(1.47 - 2.05)	<0.001***	0.98(0.81 - 1.19)	0.864
**Internet**					
No		1		1	
Yes		0.63(0.54 – 0.73)	<0.001***	0.65(0.53 – 0.78)	<0.001***
**Reading newspaper or magazine**					
No		1			
Yes		0.73(0.54 – 0.99)	0.041*	0.55(0.36 – 0.83)	0.004**
**Place of delivery**					
Other		1			
Public		0.76(0.60 – 0.96)	0.022*	0.55(0.41 – 0.74)	<0.001***
Private		0.51(0.32 – 0.81)	0.004**	0.16(0.08 -0.31)	<0.001***
NGO/Religious		2.99(1.36 - 6.58)	0.007**	1.04(0.37-2.95)	0.933
Home		0.97(0.41 - 2.32)	0.951	0.94(0.21 - 4.23)	0.939
**Place of vaccination**					
Outreach/Campaign		1		1	
Public		1.79(1.15 - 2.77)	0.009**	1.85(0.98 - 3.50)	0.057
Private		1.60(0.57 - 4.53)	0.372	1.18(0.39 -3.54)	0.767
NGO/Religious		1.10(0.37 - 3.32)	0.858	0.92(0.20 - 4.15)	0.911

**Health insurance**					
No		1		1	
Yes		0.51(0.38 – 0.67)	<0.001***	0.43(0.31 – 0.67)	<0.001***
**visited health facility the last 12 months**					
No		1		1	
Yes		1.01(0.80 - 1.29)	0.900	0.72(0.54 – 0.97)	0.031*
**Reason not using health facility/lack of access/too far**					
No		1		1	
Yes					
**Reason not using health facility/costs too much**					
No		1			
Yes		0.22(0.09 – 0.56)	0.001**	0.41(0.14 - 1.23)	0.113
**Distance to health facility**					
Not a big problem		1		1	
Big problem		0.66(0.55 – 0.80)	<0.001***	0.76(0.59 – 0.99)	0.044*
**Time to the nearest health facility**					
<30		1		1	
One hour		0.79(0.62 - 1.02)	0.069	1.06(0.77 – 1.45)	0.703
Two hours		0.88(0.59 - 1.31)	0.526	1.04(0.63 - 1.72)	0.867
Three hours		1.07(0.55 - 2.05)	0.846	1.98(1.02 - 3.83)	0.043
>3 hours		0.81(0.37 - 1.77)	0.606	2.23(0.91 - 5.45)	0.079

Note *** = p<0.001, ** = p<0.01, * = p<0.05

Supplemental Table S8. Null Model, Model 1, and 2 of multilevel logistic regression analysis in assessing factors associated with MOVs among children aged 12-23 months in SSA.

**Variables**	**Null Model**	**Model 1**		**Model 2**	
	—	Crude OR (95%CI)	P-value	Adjusted OR (95%CI)	P-value
**Caretaker variables**					
**Women aged 15-49 years**					
15-24		1			
25-34		1.03(0.85 - 1.25)	0.786		
35-49		0.83(0.65 - 1.05)	0.114		
**Number of children under 5**					
One		1			
2-3		1.14(0.94 - 1.39)	0.183		

≥4		0.92(0.68 - 1.25)	0.602		
**Number of household member**					
1-3		1			
4-5		1.30(0.98 - 1.74)	0.072		
≥6		1.19(0.88 - 1.62)	0.246		
**Mode of transportation to the nearest health facility**					
walking		1			
car/truck/bus/taxi/boat motor		0.97(0.80 - 1.18)	0.796		
bicycle/animal-drawn/boat no motor		0.62(0.48 – 0.81)	<0.001***		
**Mother’s education**					
No education		1			
Primary		0.88(0.70 - 1.10)	0.263		
Secondary or higher		0.73(0.57 – 0.94)	0.017*		
**Father's education**					
no education		1			
primary		0.80(0.63 - 1.03)	0.084		
secondary or higher		0.85(0.66 - 1.11)	0.237		
unknown		0.98(0.65 - 1.46)	0.918		
Residence					
Urban		1			
Rural		1.67(1.12 - 1.56)	0.002**		
**Occupation**					
Not working		1			
Manual/HH/Domestic		0.77(0.60 - 1.00)	0.055		
Sales		0.58(0.46 – 0.72)	<0.001***		
Agriculture		0.50(0.35 – 0.72)	<0.001***		
Pro/Tec/Man/Cler/Ser/Other		0.95(0.74 - 1.21)	0.666		
**Wealth index quintile**					
Poorest		1			
Second		0.91(0.71 - 1.16)	0.442		
Middle		0.82(0.62 - 1.07	0.147		
Fourth		0.69(0.50 – 0.94)	0.018*		
Richest		0.75(0.51 – 1.10)	0.141		
**Religion**					
Animist/Traditionalist/No religion		1			
Muslim		1.20(0.83 - 1.74)	0.323		
Catholic		1.36(0.91 - 2.04)	0.138		
Protestant		0.88(0.58 - 1.35)	0.562		
Other Christian		1.33(0.92 - 1.93)	0.130		
**Listening to the radio**					
No		1			
Yes		0.59(0.50 – 0.70)	<0.001***		
**Watching television**					
No		1			
Yes		0.84(0.68 - 1.03)	0.091		
**Internet**					
No		1			
Yes		0.84(0.67 - 1.04)	0.106		

**Health system variables**					
**Place of delivery**					
Home				1	
Public				0.32(0.20 – 0.53)	<0.001***
Private				0.78(0.21 - 2.91)	0.709
NGO/Religious				9.09(0.83 – 15.49)	0.070
Other				0.28(0.04 - 1.76)	0.175
**Place of vaccination**					
Campaigns/Outreaches				1	
Public				0.87(0.43 - 1.78)	0.710
Private				0.59(0.12 - 2.87)	0.519
NGO/Religious				0.62(0.08 - 4.90)	0.647
**Health insurance**					
No				1	
Yes				0.40(0.24 – 0.66)	<0.001***
**visited health facility the last 12 months**					
No				1	
Yes				0.85(0.59 - 1.23)	0.388
**Reason not using health facility/costs too much**					
No				1	
Yes				2.60(0.53 - 12.68)	0.235
**Distance to health facility**					
Not a big problem				1	
Big problem				1.08(0.74 - 1.58)	0.680
**Time to the nearest health facility**					
<30 minutes				1	
One hour				1.34(0.91 - 1.96)	0.138
Two hours				1.37(0.73 - 2.58)	0.323
Three hours				2.25(0.73 - 6.97)	0.159
>3 hours				0.41(0.10 - 1.58)	0.196

Note *** = p<0.001, ** = p<0.01, * = p<0.05

Supplemental Table S9. Predicted posterior mean of MOVs in children aged 12-23 months using Model 3 in SSA.

Country	Average marginal effect	Standard error	95CI%
Benin	0.01	0.09	-0.17 to 0.19
Burkina-Faso	0.01	0.09	-0.19 to 0.17
Chad	-0.00	0.09	-0.19 to 0.18
Comoros	-0.00	0.09	-0.19 to 0.18
Cote d'Ivoire	0.008	0.09	-0.19 to 0.18
Eswatini	0.02	0.09	-0.19 to 0.18
Gabon	-0.19	0.09	-0.20 to 0.17
Ghana	0.01	0.09	-0.17 to 0.19
Gambia	0.03	0.09	-0.15 to 0.21
Kenya	0.06	0.09	-0.11 to 0.24
Liberia	-0.02	0.09	-0.21 to 0.16
Madagascar	0.04	0.09	-0.22 to 0.14
Malawi	-0.05	0.09	-0.23 to 0.13
Mozambique	0.03	0.09	-0.14 to 0.21
Nigeria	0.02	0.09	-0.15 to 0.19
Rwanda	-0.00	0.09	-0.19 to 0.18
Sao Tome and Principe	-0.00	0.09	-0.20 to 0.18
Senegal	0.04	0.09	-0.13 to 0.22
Sierra-Leone	0.00	0.09	-0.18 to 0.19
Tanzania	-0.02	0.09	-0.20 to 0.16
Zimbabwe	0.01	0.09	-0.19 to 0.17

Supplemental Table S10. Model-predicted probability of uncorrected, corrected, and some corrected MOVs in multinomial logistic regression analysis using key variables

	Margin value (95%CI)	P-Value
**0. Uncorrected MOVs**		
**Mother's education**		
No education	0.48(0.45 – 0.50)	<0.001
Primary	0.38 (0.35 – 0.40)	<0.001
Secondary or higher	0.43 (0.41 – 0.46)	<0.001
**Wealth**		
Poorest	0.47(0.44 – 0.50)	<0.001
Second	0.43(0.40 – 0.46)	<0.001
Middle	0.42(0.39 – 0.46)	<0.001
Fourth	0.43(0.39 – 0.46)	<0.001
Richest	0.38(0.34 – 0.42)	<0.001
**Listening radio**		
No	0.46(0.44 – 0.48)	<0.001

Yes	0.39(0.37 – 0.41)	<0.001
**Watching television**		
No	0.39(0.38 – 0.41)	<0.001
Yes	0.47(0.45 – 0.50)	<0.001
**Internet**		<0.001
No	0.41(0.39 – 0.42)	<0.001
Yes	0.49(0.47 – 0.52)	<0.001
**Insurance**		
No	0.41(0.40 – 0.43)	<0.001
Yes	0.53(0.49 – 0.57)	<0.001
**1. Corrected MOVs**		
**Mother's education**		
No education	0.37(0.34 – 0.39)	<0.001
Primary	0.46(0.43 – 0.46)	<0.001
Secondary or higher	0.44(0.41 – 0.47)	<0.001
**Wealth**		
Poorest	0.37(0.33 – 0.40)	<0.001
Second	0.42(0.39 – 0.45)	<0.001
Middle	0.43(0.40 – 0.46)	<0.001
Fourth	0.44(0.41 – 0.47)	<0.001
Richest	0.47(0.43 – 0.51)	<0.001
**Listening radio**		
No	0.38(0.36 – 0.40)	<0.001
Yes	0.46(0.44 – 0.49)	<0.001
**Watching television**		
No	0.45(0.43 – 0.47)	<0.001
Yes	0.38(0.36 – 0.40)	<0.001
**Internet**		
No	0.44(0.43 – 0.46)	<0.001
Yes	0.36(0.33 – 0.39)	<0.001
**Insurance**		
No	0.43(0.42 – 0.45)	<0.001
Yes	0.33(0.30 – 0.37)	<0.001
**2. Some corrected MOVs**		
**Mother's education**		
No education	0.15(0.14 – 0.17)	<0.001
Primary	0.16(0.14 – 0.18)	<0.001
Secondary or higher	0.13(0.11 – 0.14)	<0.001
**Wealth index**		
Poorest	0.16(0.14 – 0.18)	<0.001
Second	0.15(0.13 – 0.17)	<0.001
Middle	0.14(0.12 – 0.16)	<0.001
Fourth	0.13(0.11 – 0.15)	<0.001
Richest	0.14(0.12 – 0.17)	<0.001
**Listening radio**		

No	0.15(0.14 – 0.17)	<0.001
Yes	0.14(0.12 – 0.15)	<0.001
**Watching television**		
No	0.15(0.14 – 0.16)	<0.001
Yes	0.14(0.13 – 0.16)	<0.001
**Internet**		
No	0.15(0.14 – 0.16)	<0.001
Yes	0.15(0.12 – 0.17)	<0.001
**Insurance**		
No	0.15(0.14 – 0.16)	<0.001
Yes	0.14(0.11 – 0.16)	<0.001

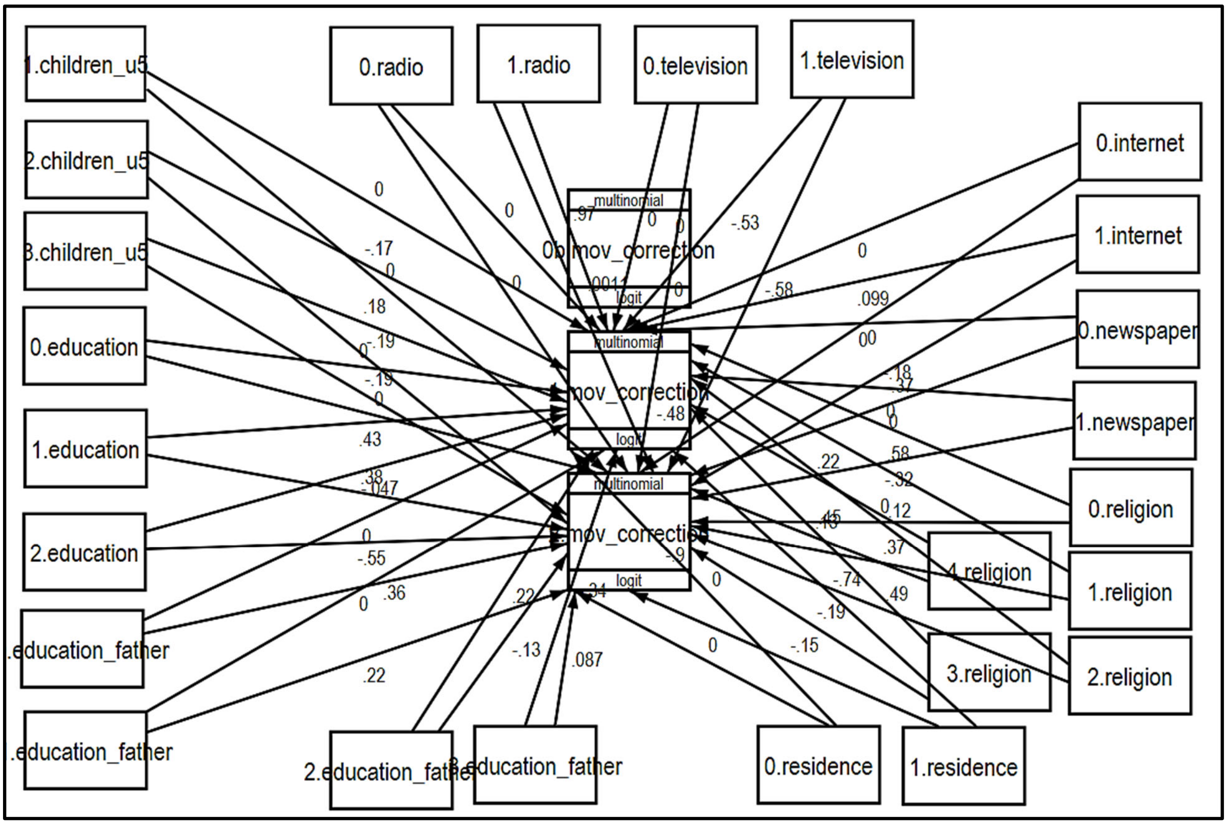

Supplemental Figure S1. Model 1 using Generalized Structural Equation Modelling (GSEM) for multilevel multinomial logistic regression analysis

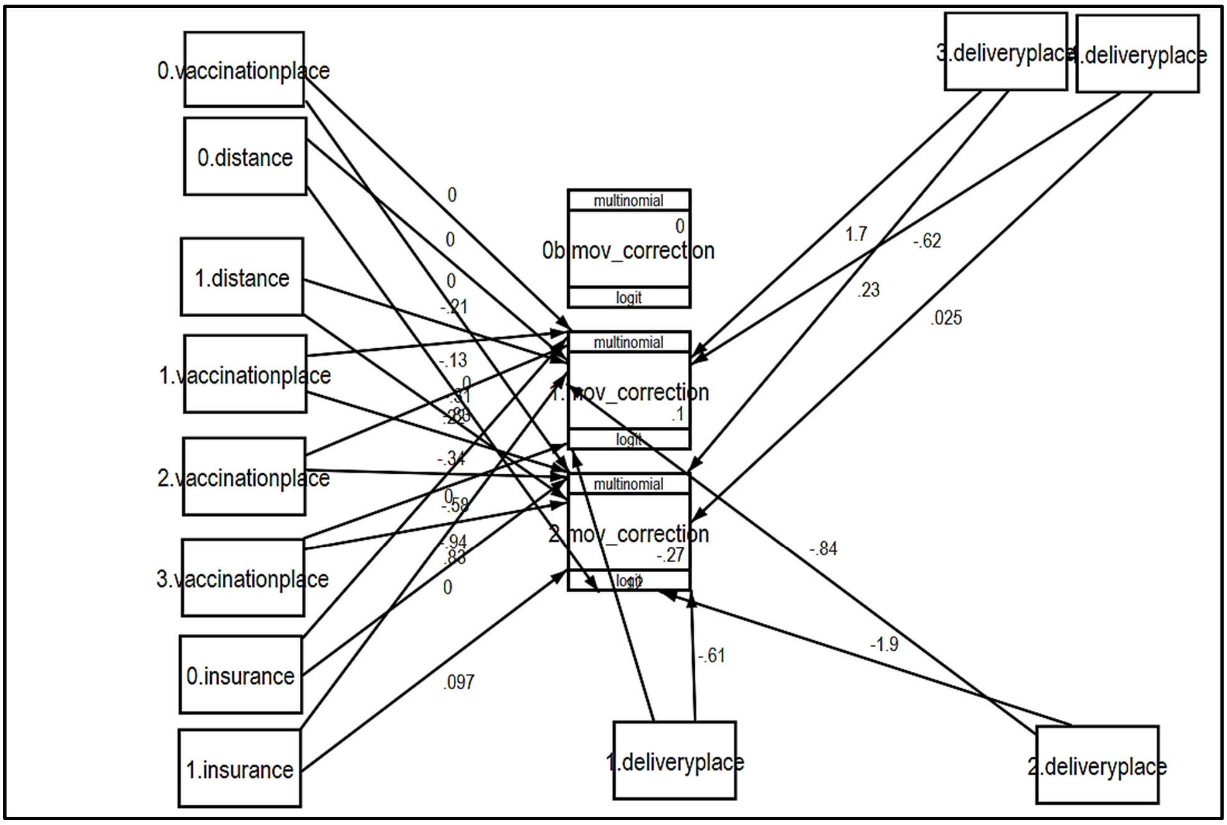

Supplemental Figure S2. Model 2 using Generalized Structural Equation Modelling (GSEM) for multilevel multinomial logistic regression analysis

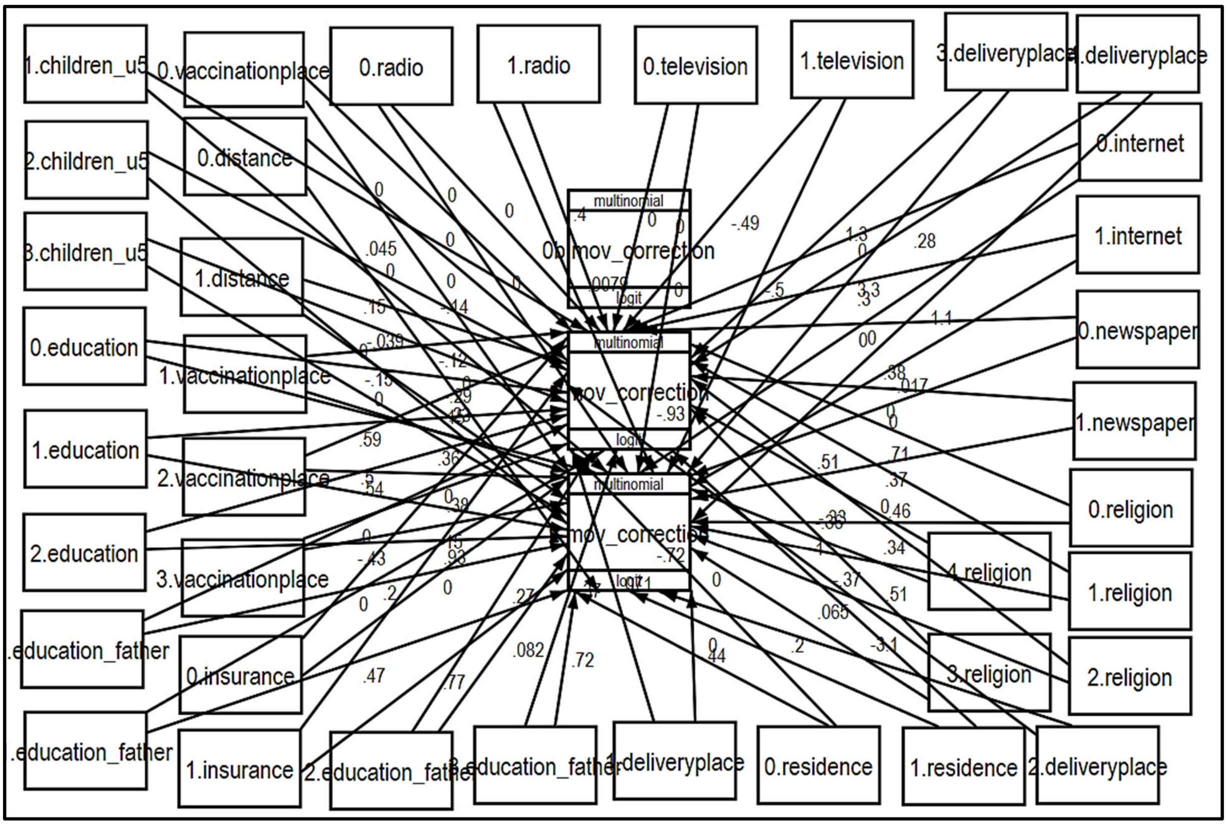

Supplemental Figure S3. Model 3 using Generalized Structural Equation Modelling (GSEM) for multilevel multinomial logistic regression analysis
